# Big data empowerment: Digital transformation and governance of minority shareholders: Evidence from China

**DOI:** 10.1371/journal.pone.0302268

**Published:** 2024-04-16

**Authors:** Xinhao Hou, Yao Tang

**Affiliations:** Business School, University of International Business and Economics, Beijing, China; Ovidius University of Constanta: Universitatea Ovidius din Constanta, ROMANIA

## Abstract

Based on the analysis of data from listed enterprises in China between 2011 and 2022, we investigate the influence of digital transformation on the governance efficiency for minority shareholders. The results show that the extent of digital transformation exert a negative effect on the agency costs incurred from related-party transactions. The mechanism examination elucidates that digital transformation augments the governance efficiency for minority shareholders by boosting attendance at shareholders’ meetings and enhancing the exit threat for minority shareholders. Subsequent analysis reveals that non-state-owned enterprises, compared to state-owned enterprises, exhibit a more pronounced effect in diminishing the second type of agency costs through digital transformation. Furthermore, the impact of digital transformation in curtailing agency costs is more significant in the eastern regions than central and western regions. The better the equity checks and balances in listed enterprises, the more effective digital transformation is in reducing agency costs. This study offers valuable insights for bolstering the governance capacity of minority shareholders in the context of digital transformation.

## 1. Introduction

For a long time, the protection of minority shareholders’ rights and governance issues have been one of the persistent challenges troubling the capital markets in China. Minority shareholders typically refer to those public shareholders in listed enterprises other than the controlling shareholders and institutional investor shareholders. In China, investor protection mechanisms have imperfections, and share ownership is highly concentrated. These issues lead to frequent infringements on the rights and interests of minority shareholders by controlling shareholders and corporate executives. Consequently, minority shareholders find themselves in a long-term “naturally disadvantaged” position in the governance process [1, 2]. Most minority shareholders lack enthusiasm in participating in shareholders’ meetings, and their attention to the company’s operational results and daily business activities is far less than that of controlling shareholders. Therefore, under the traditional corporate governance framework, the efficiency of minority shareholders’ participation in corporate governance has not received effective attention. Meanwhile, the indifference of minority shareholders towards participating in corporate governance also costs them dearly. On one hand, the lack of supervision and restraint from minority shareholders fuels the motivation of large shareholders to hollow out listed enterprises, plundering the company’s resources and assets, thereby harming the interests of minority shareholders [3]. On the other hand, the management may take advantage of this to engage in opportunistic behaviors, with shareholders bearing the agency costs arising from managerial self-interest [4]. The protection of minority shareholders’ rights and the effectiveness of their participation in corporate governance remain pressing issues in the practice of capital markets in China.

As the digital economy develops rapidly, the role of stakeholders becomes more significant, and the focus on information disclosure grows. Additionally, shareholders now have more channels to access information and participate in corporate governance. As a result, minority shareholders are presented with increased opportunities and methods to engage in corporate governance. Chinese capital market legal framework has seen significant enhancements. Alongside this, there’s a rise in minority shareholder rights awareness and a digital transformation occurring. These changes have led to new online-engagement practices. These practices are altering the previously disadvantaged position of minority shareholders in corporate governance. The governance of minority shareholders is having a significant and lasting impact on the governance practices of listed enterprises in China. These arouse scholars’ attention to the efficiency of minority shareholder participation in corporate governance. A series of international literature and case studies have pointed out the reshaping of corporate governance in the era of digital economy. For example, a modern view of stewardship in a digital economy has been proposed, emphasizing truthfulness, transparency, trust, and technological equity as the key pillars of virtuous stewardship in corporate governance. Additionally, digital platforms have enabled minority shareholders to participate in online voting, thereby playing a crucial supervisory role on the management, improving the level of internal control, and reducing the overconfidence of the management. Furthermore, the engagement of minority shareholders through digital channels, as evidenced by the minority shareholder activism in China, has been found to improve corporate social responsibility performance. Research on whether digital transformation in traditional industries can improve accounting quality as well as corporate governance has also been conducted. Considering technological, industrial revolutions, and national digital strategies, an empirical study of digital transformation’s impact on minority shareholder governance is pivotal. This includes assessing changes in role, governance mechanisms, and efficiency. Such inquiry holds significant reference for empowering corporate governance by minority shareholders, safeguarding their rights, promoting high-quality development, and ultimately enhancing the capital market environment.

Our study, employing data from Chinese A-share listed enterprises from 2011 to 2022 as the sample, empirically examines the impact of digital transformation on the governance efficiency of minority shareholders. The study finds that the extent of digital transformation has a negative effect on the level of related transactions in listed enterprises, indicating that digital transformation can reduce the agency costs brought about by related transactions, thereby enhancing the governance efficiency of minority shareholders. Furthermore, we discover that digital transformation enhances the governance efficiency of minority shareholders in two ways. First, it leads to increased attendance of minority shareholders at shareholders’ meetings. Second, it amplifies the influence of minority shareholders, as indicated by their potential to exit the company. The primary contributions of our study include the following aspects:

First, our research enriches the study on the governance efficiency of minority shareholders in the context of the digital economy era. Previous studies mostly based their discussions on the governance efficiency of minority shareholders within corporate governance structures where large shareholders have excess control rights, such as the “pyramidal” ownership structures. However, with the advent of the digital economy, the gradual dispersion of equity in listed enterprises in China, and the growing emphasis on “Environmental, Social and Governance” disclosures, minority shareholders have become significant entities in the governance of listed enterprises. Through empirical examination, we investigate the impact of digital transformation from a big data empowerment perspective on the governance efficiency of minority shareholders, corroborating the findings of *Chen and Srinivasan (2023)* regarding the evolution of external governance modes of listed enterprises against the backdrop of digital transformation [5]. This provides a supplementary lens to the corporate governance research in the digital economy era.

Second, our research broadens the understanding of the economic consequences of digital transformation. The current literature mainly explores digital transformation’s effects on corporate performance, market valuation, R&D innovation, and investment efficiency, overlooking its impact on listed enterprises’ governance. There is a limited number of empirical studies examining how digital transformation influences corporate governance. Focusing on minority shareholder governance issues, we explore the relationship between digital transformation and minority shareholder governance, offering corroborative evidence from China. This contributes to the theoretical research by *Zhu (2019)* and others, on how digital technologies affect corporate governance [6]. This contributes a supplementary perspective to the related research on the economic repercussions of corporate digital transformation.

The rest of our study is structured as follows: Section 2 presents the literature review. Section 3 presents the theoretical analysis and research hypothesis. Section 4 details the research design. Section 5 provides the regression results and further analysis. Section 6 concludes with research findings and implications.

## 2. Literature review

### 2.1 Minority shareholders’ participation in corporate governance

The governance of minority shareholders gained attention in the 1990s, driven by the issues of concentrated ownership among large shareholders and their resultant exploitation of minority interests. Studies, including *La Porta et al*. *(1999)*, identified a trend of high equity concentration in developed countries, causing conflicts between large and minority shareholders and raising concerns about minority shareholder governance globally [1, 2]. In China, the evolution of capital markets and policies has underscored the importance of protecting minority shareholders from controlling shareholders’ exploitations, such as through pyramidal structures and crossholdings.

Research on minority shareholder governance focuses on their motivations for involvement in corporate governance—mainly to prevent rights infringements by large shareholders and promote transparency [7, 8], the mechanisms they use, including voting rights and share selling, and the effects of their participation [9, 10]. Technological and legal advancements have empowered minority shareholders with better information access, encouraging their active participation. While some studies highlight the benefits of such involvement, like reduced appropriation of interests by large shareholders and enhanced long-term company performance [11–14], others caution against potential negative impacts due to information asymmetry and the risk of hindering a company’s long-term growth [15]. This body of work underscores the complexity of minority shareholder participation in corporate governance, reflecting a balance between safeguarding their interests and ensuring the sustainable development of corporations.

### 2.2 Digital transformation

Recent advances in digital technologies, driven by IT giants such as Microsoft and Google, have transformed society, affecting communication, industrial structures, consumer behaviors, and business models. As a result, many non-IT enterprises are embracing digital tools like big data, IoT, cloud computing, AI, and blockchain to innovate and optimize their operations, a process known as Digital Transformation [16, 17]. This transformation is essential for businesses aiming to capitalize on new opportunities, gain competitive advantages, and foster technological innovation [18]. At the micro-level, it offers firms long-term benefits and increased competitiveness, while at the macro-level, it facilitates a shift towards digital economic systems, enhancing societal productivity and efficiency.

Research on digital transformation primarily explores its economic implications, showing that adopting digital technologies can automate production, boost productivity, and improve organizational and investment efficiency [19–21]. It also enables precise innovation efforts, reducing the risks associated with R&D [22], and expands enterprises’ growth potential, attracting investors and raising market value [23]. Despite the extensive investigation into these economic benefits, studies on digital transformation’s impact on organizational change, particularly corporate governance, remain limited. A few works have theoretically and through case studies explored how digital technology reshapes corporate governance, affecting the external governance environment and optimizing shareholder meetings to curb managerial opportunism [6, 24]. Yet, empirical evidence supporting these findings is scant, highlighting a gap in research that needs addressing to understand fully the relationship between digital transformation and corporate governance.

## 3. Theoretical analysis and research hypotheses

### 3.1 The governance effects on minority shareholders of digital transformation

In the long-term practice of Chinese capital market, minority shareholders have faced several negative factors. These include their smaller scale and lesser shareholdings. Additionally, there’s low transparency in the information disclosure of listed enterprises. Minority shareholders also experience restricted access to information and participation in corporate governance. As a result, their enthusiasm for participating in corporate governance is low, and their overall impact on enterprises is minor, remaining in a state of insignificance for a long time [7]. However, recent trends in the capital market have led to the decentralization of shareholdings. Alongside this, there has been a rise in digital technologies like big data and the internet. These changes enable shareholders to access a wide range of internal and external information about listed enterprises through public channels. This information is then reflected in stock prices, exerting profound impacts on listed companies [23, 25]. This provides important opportunities for minority shareholders to change their disadvantaged position and actively participate in corporate governance. The primary motivation for minority shareholders to engage in corporate governance is to prevent misconduct by major shareholders. This misconduct includes emptying listed enterprises through related transactions, transfer payments, and other forms of interest encroachment. Such participation aims to ensure stable investment returns for minority shareholders [24, 26].

During the process of digital transformation, companies generate a massive amount of big data that reflects their operational information. The effects on minority shareholders are twofold: On one hand, as digital transformation becomes a dominant trend in current Chinese enterprises, the board of directors and management are more motivated to explain and disclose in annual reports the big data generated during the post-digital transformation production and operation processes. This aims to attract the attention of minority shareholders and other external investors for financing. Minority shareholders are more likely to focus on these enterprises undergoing digital transformation in hope of gaining investment returns, thereby increasing their motivation to participate in corporate governance. On the other hand, the big data from the process of digital transformation is more objective and realistic, and more readily accessible by minority shareholders. As a result, the enhanced transparency of enterprise information further boosts their motivation to partake in governance and safeguard their rights and interests. Therefore, the impact of digital transformation on minority shareholder governance is reflected in reducing the degree of interest encroachment by major shareholders on minority shareholders, i.e., reducing the agency costs among shareholders [4]. Based on the above analysis, we propose Hypothesis 1:

Hypothesis 1: Digital transformation can reduce the agency costs, enhancing the governance effect of minority shareholders.

### 3.2 The mechanisms of digital transformation impacting minority shareholder governance effectiveness

*Harris (2010)* discussed the mechanisms available to minority shareholders for participating in corporate governance. These mechanisms include participating in shareholders’ meeting resolutions and actively exercising voting rights. Additionally, minority shareholders can form exit threats by selling company shares when their interests are damaged. These actions serve to restrain improper decisions by controlling shareholders in two ways [27]. In Chinese capital market, minority shareholders have long been at a disadvantage. Factors contributing to this include a smaller shareholding ratio and information asymmetry. However, several developments are changing this scenario. The growth of the digital economy and the implementation of digital transformation are key factors. Additionally, the rise of shareholding decentralization and the advent of online voting are playing a role. These changes have gradually increased the voice of minority shareholders. They have also broadened channels for information acquisition and enhanced governance effectiveness through voting rights and exit threats.

First, from the perspective of exercising voting rights, on one hand, the advent of digital transformation has improved the transparency of internal and external information of enterprises, bringing information advantages to all shareholders [28]. In traditional models, due to delays and asymmetry in information transmission, minority shareholders often could not grasp the true operational status of the company in a timely manner, resulting in damaged interests. Modern digital technologies, especially big data, cloud computing, and blockchain technology, ensure the timeliness, completeness, and immutability of information, enabling all shareholders to equally access company information, thus providing more decision references for minority shareholders [6]. Minority shareholders can grasp the macroeconomic background and business dynamics of the company through broader information channels, significantly improving company information transparency and alleviating the information disadvantages of minority shareholders [29]. On the other hand, with the enhancement of big data processing capabilities and digital endowment of minority shareholders, they can utilize advanced digital technologies to enhance their information processing and analysis capabilities, optimize behavioral decisions based on data, and make leaps and bounds in participating in corporate governance with professionalism [16, 26, 28]. The increased information transparency caused by digital technologies, along with the increased efficiency of information acquisition and utilization by minority shareholders, will further motivate minority shareholders to actively exercise voting rights in shareholders’ meetings to ensure the implementation of their will and protect their legitimate rights. Therefore, digital transformation will increase the extent of minority shareholder participation in corporate governance by exercising voting rights.

Second, from the perspective of exit threats, the vast amount of big data generated from digital transformation has multiple uses. Not only can it be utilized by the enterprise itself, but it’s also accessible to customers and investors. This data spreads within their respective networks. In recent years, the growth of social media and self-media platforms has amplified this effect. Platforms like Twitter in the US, and Weibo, WeChat, Guba, and Xueqiu in China, have played a significant role. They have brought a wide and relatively unfamiliar group of listed enterprises into the public eye, subjecting them to social supervision. Coupled with big data technology, precise information delivery to investors has been realized [30]. When listed enterprises face public relations crises or litigation due to controlling shareholders seeking personal gains, minority shareholders often discuss these issues extensively on social media platforms like Twitter and Weibo and form strong exit threats by selling shares. This will be exposed to the public through social media platforms, causing a significant decline in the stock price of listed enterprises. Following the release of the “Interpretation on Several Issues Concerning the Application of Law in Handling Criminal Cases of Defamation Using Information Networks” by China in September 2013, the listed enterprises have had to pay attention to their internet information environment, making discussions about harming minority shareholder interests on internet and social media platforms more deterrent to controlling shareholders [30]. Therefore, while digital transformation reduces information asymmetry within and outside enterprises, it also amplifies the effect of exit threats by minority shareholders through networks and social media. Therefore, digital transformation will increase the extent of minority shareholder participation in corporate governance using exit threats. Based on the above analysis, we propose Hypothesis 2:

Hypothesis 2: Digital transformation can increase the extent to which minority shareholders participate in governance through exercising voting rights and exit threats, thereby enhancing the governance effect of minority shareholders.

## 4. Sample selection and research design

### 4.1 Research samples

We use all enterprises listed on the Shanghai and Shenzhen A-share market in China from 2011 to 2022 as our research sample. According to the research needs, we refine this sample in several steps to meet our research requirements. First, we exclude enterprises listed as ST and *ST. ST companies and *ST companies are labels used to denote enterprises that have experienced operational losses for two and three consecutive years, respectively, posing a risk of delisting. These entities commonly encounter severe operational and financial challenges, with some even undergoing bankruptcy proceedings. Such circumstances may prevent them from adhering to standard accounting policies. Consequently, the data in their financial reports often lacks referential value or is distorted by outliers. For these reasons, the study excludes these samples. Next, we remove any observations with missing variables. Then, to reduce the impact of outliers and ensure value stability across variables, we Winsorize all continuous variables at the 1% and 99% levels and apply Z-score normalization. This process results in a final sample of 3,336 listed enterprises, encompassing 31,649 observations.

Our data is derived from the following methods and databases: The system of variables for digital transformation is generated from text analysis of listed enterprises’ annual reports using PyTorch, Jieba, Scikit-learn and other Python scripts, as well as from the Wind and China Stock Market & Accounting Research (CSMAR) databases, and from manual analysis and collection of documents such as listed enterprises’ annual reports, company articles, and prospectuses. The variables for minority shareholder governance are sourced from analyzing and statistically processing the situations of shareholders’ meetings disclosed by listed enterprises using Python scripts, as well as from the CSMAR database. The relevant data concerning the local digital economic development level is obtained from the Wind database. Other financial and trading data of listed enterprises are sourced from the Wind and CSMAR databases.

### 4.2 Variable definitions

#### 4.2.1 Dependent variable: Agency cost (*RPT*)

Agency costs among shareholders are also known as the conflict of interest between major and minority shareholders. This refers to a scenario where controlling shareholders pursue personal gains. They do this through related transactions, capital occupation, and other actions. Such activities end up harming the interests of minority shareholders. According to existing literature, many studies measure the agency costs based on two dimensions: the extent of capital occupation and the extent of engagement in related transactions by listed enterprises. However, with the China Securities Regulatory Commission intensifying the regulation on capital occupation behaviors in 2003, it has become difficult for controlling shareholders to continue seeking personal gains through direct capital occupation, and they have turned to related transactions to realize private benefits more. Therefore, referring to the studies by *Jiang et al*. *(2015)* and *Brockman et al*. *(2019)*, We use the variable *RPT*, which is the ratio of the total amount of related transactions to total assets, to measure agency costs [31, 32].

#### 4.2.2 Independent variable: Digital transformation (*DT*)

In the process of digital transformation, big data will be generated in various links such as strategy, production, and business processes. Enterprises usually extract and utilize this big data to empower production decision-making and operational management, hence the process of digital transformation can be regarded as a process of empowering with big data. In recent years, many studies have adopted the quantitative statistical method of keyword frequency analysis on digital technology from the annual reports of listed enterprises to measure the level of digital transformation [5]. However, the keywords disclosed in the annual reports of listed enterprises only reflect the enterprise’s strategic focus on digital technology and corresponding plans, and do not necessarily indicate that the enterprise has truly taken actions toward digital transformation. Therefore, it is necessary to supplement and improve the existing measurement methods from business and information perspectives. For shareholders, the process of digital transformation involves enhancing their understanding of big data. This is crucial for better utilizing the various big data generated in enterprise production and operational processes. By doing so, they can strengthen supervision and restraint on enterprise management. This effort aligns with the governance objectives of the shareholders. From the perspective of big data empowerment, digital transformation offers a core advantage. This advantage is the information benefit stemming from a large amount of operational activity data generated by enterprises. Additionally, digital transformation enhances shareholders’ abilities to obtain information through various channels. Based on this, our study takes big data empowerment, namely the information advantage and enhancement of shareholders’ big data utilization capabilities brought by digital transformation as dimensions, and drawing on the method of *Sarma (2012)*, we use multi-dimensional principal component analysis to construct the *DT* variable [33]. Details are as follows:

*A*. *Information Advantage*. With enterprises actively adopting cutting-edge digital technologies such as big data, cloud computing and AI, the amount of big data generated in areas of performance disclosure, production modes, and customer management has significantly increased compared to the past. Minority shareholders are no longer passive recipients of information; they can proactively acquire this rich big data, and through in-depth analysis and fine refinement, provide clearer and stronger support for all shareholders in decision-making and corporate governance activities. This new data-driven approach grants minority shareholders an unprecedented information advantage. To better understand this phenomenon, our study measures the information advantage of digital transformation from four sub-dimensions: corporate strategy, financial accounting, infrastructure, and business model.

First, in terms of corporate strategy, our study refers to the method of *Li et al*. *(2020)* and *Chen and Srinivasan (2023)*, constructing digital keywords in six aspects: “big data, artificial intelligence (AI), the Internet of Things, cloud computing, virtual reality/VR/AR, and blockchain,” further enriching the quantity of keywords through machine learning methods [5, 34]. We initially constructed a seed lexicon, encompassing the principal keywords of six dimensions of digital transformation–Big Data, Artificial Intelligence, Internet of Things, Cloud Computing, Virtual Reality/VR/AR, and Blockchain. Subsequently, this study utilized the annual reports from the recent five years of listed companies as original documents, and accomplished vectorization of words through word embedding technology. Lastly, by computing the similarity of word vectors, we extracted the top 50 words most relevant to each seed word, and manually assessed whether they belong to the digital transformation keywords. Beyond machine learning methods, we further refined our dictionary by drawing on the expertise of other specialists. We referred to the whitepapers issued by China Academy of Information and Communications Technology, including “China Digital Economy Development Whitepaper”, “Big Data Whitepaper”, “Artificial Intelligence Core Technology Industry Whitepaper”, “Internet of Things Whitepaper”, “Cloud Computing Development Whitepaper”, “Virtual Augmented Reality Whitepaper”, and “Blockchain Whitepaper” to enhance the dictionary. Through the efforts, this study eventually constructed a digitalization dictionary comprising six modules and 119 keywords, which are shown in Table 1. After constructing the keyword list, our study employs a Python script to perform word frequency statistics on the term of digitalization keywords in the annual reports of listed enterprises, where a higher frequency indicates a greater strategic emphasis on digitalization.

**Table 1:** The dictionary of digitalization keywords.

Modules	
Big Data	Big Data, Massive Data, Distributed Computing, Data Integration, Metadata, Data Modeling, Data Standard Management, Data Quality Management, Data Assets, Data Mart, Data Warehouse, Data Labeling, Master Data Discovery, Data Standard Application, Graph Analysis, Graph Data, Graph Structured Data, Graph Modeling, Graph Computing, Knowledge Graph, BI Tools, Business Intelligence, Data Visualization, Data Mining, Unstructured Data, Heterogeneous Data, Hadoop, Spark
Artificial Intelligence (AI)	Artificial Intelligence, AI, Machine Learning, Deep Learning, Reinforcement Learning, Transfer Learning, Adversarial Learning, Supervised Learning, Multimodal Learning, Knowledge Engineering, Neural Networks, Pre-trained Models, Autonomous Driving, Data Annotation, Speech Recognition, Image Recognition, Machine Translation, Robotics, Computer Vision, Natural Language Processing, Intelligent Recommendation, Face Recognition, Image Recognition, Intelligentization, Intelligent Manufacturing, Intelligent Finance, Intelligent Healthcare, Intelligent Security, Intelligent Transportation, Intelligent Healthcare, Smart Cities, Intelligent Agriculture
Internet of Things (IoT)	Internet of Things, IoT, Smart Home, Wearable Devices, Sensors, eSIM, RFID, Electronic Tags, Edge Computing, Edge Terminals, Edge Gateway, Edge Controllers, Photovoltaic Cloud Network, Vehicle Networking, Industrial Internet of Things, Industrial Internet.
Cloud Computing	Cloud Computing, Cloud Platform, Cloud Services, Public Cloud, Private Cloud, Hybrid Cloud, Cloud Endpoint, IaaS, PaaS, SaaS, Cloud-Edge Collaboration, Cloud-Native, Container Technology, Containerization, Microservices, DevOps
Virtual Reality (VR/AR)	Virtual Reality, Augmented Reality, VR, AR, Human-Computer Interaction, Perceptual Interaction, Near-Eye Display, Rendering Computation, Cloud Rendering, Gaze Point Technology, Gaze Point Optics, Gaze Point Rendering, Eye Tracking, Gesture Tracking, Head-Mounted Display
Blockchain	Blockchain, Consortium Chain, Public Chain, Public Chain, Private Chain, Digital Currency, Bitcoin, BaaS, Cryptographic Algorithm, Peer-to-Peer Network, Consensus Mechanism, Smart Contract

Second, in the domain of financial accounting, an important indication of digitalization is the enterprise’s utilization of ERP accounting systems. Hence, referring to the method of *Dorantes et al*. *(2013)*, our study utilizes Python programs and manual collection to perform text analysis on documents like listed enterprises’ annual reports, prospectuses, and third-party tracking rating reports, to determine whether listed enterprises are employing ERP accounting systems [35]. Specifically, we initially employed keywords such as “ERP,” “Enterprise Resource Planning,” and names of ERP vendors to locate publicly disclosed documents like annual reports of listed companies, preliminarily screening out companies that utilize ERP systems. Subsequently, by reading the relevant disclosure documents, we determined whether the listed companies truly adopted ERP systems and the time of adoption, with related keywords including “started using”, “put into operation”, “completed”, “developed” and “launched”. We establish dummy variables to measure whether the sample companies adopt ERP systems, serving as a sub-variable for the digital transformation in the financial accounting dimension.

Third, in the aspect of infrastructure, our study follows digital keywords to filter the names of infrastructure projects invested in by enterprises, summarizing the total amount of project investments. Last, in terms of business models, our study conducts text analysis on public announcements such as listed enterprises’ annual reports, locating keywords like “smart manufacturing,” “CRM system/customer information management system,” “platform economy” and “sharing economy” to determine whether enterprises are applying digital technology in their business models and undergoing transformation.

*B*. *Shareholder Capability*. The mastery and application capabilities of shareholders regarding digital resources largely determine whether the information advantage in the digital transformation of enterprises can be fully unleashed. The capability of minority shareholders—from acquiring, parsing, to applying the big data generated through digitalization—bears on their influence and role in corporate governance. To some extent, shareholders’ digital capabilities become a crucial indicator to measure the success of digital transformation. Based on this, our study measures the change in shareholder capability during digital transformation from three sub-dimensions: shareholder digital background, fintech, and online attention.

First, concerning the shareholder digital background, our study employs the natural logarithm of the number of directors with information and technology backgrounds among the directors appointed by non-controlling shareholders to measure whether the directors appointed by minority shareholders have sufficient capability to handle big data. Second, in the aspect of fintech, referring to the research of *Tang et al*. *(2020)*, our study utilizes the Digital Financial Inclusion Index at the provincial level compiled by Peking University’s Digital Finance Research Center, employing the digital financial inclusion index of the region where the listed company is located to measure the overall fintech level of the area [36]. This reflects to what extent minority shareholders can utilize fintech to handle big data and participate in governance. Third, regarding online attention, following the studies of *Sun et al*. *(2020)*, our study employs China’s Baidu (similar to Google) search index and comments count on Guba (similar to Reddit or Seeking Alpha) to measure the extent to which minority shareholders in China utilize online platforms to handle big data and the reactions triggered [37].

The specific definitions and descriptive statistics of the indicators for the above two dimensions are shown in Table 2.

**Table 2:** Measurement of the digital transformation variable.

Primary Demention	Secondary Demention	Variable Label	Variable Type	Mesurement Method
Information Advantage	Corporate strategy	*DT_CS*	Continuous variable	The value of firms’ annual report digital keywords frequency count
	Financial Accounting	*DT_FA*	Dummy variable	Adoption of ERP Accounting System
	Infrastructure	*DT_INF*	Continuous variable	The value of total investment in digital infrastructure projects based on digital keywords
	Business model	*DT_BM*	Dummy variable	Adoption of smart manufacturing, CRM (customer information management) system, platform economy/shared economy model
Shareholder capability	Shareholder digital background	*DT_SDB*	Continuous variable	The ratio of directors with information and technology background among non-controlling shareholder-appointed directors to total number of directors
	Fintech	*DT_FIN*	Continuous variable	Digital financial inclusion index of the province where the listed company is located
	Internet attention	*DT_BAIDU*	Continuous variable	The value of Baidu Search Index
		*DT_GUBA*	Continuous variable	The value of Guba comments amount

Upon establishing the indicator system for digital transformation, we carried out dimensionless processing and principal component analysis on the indicators separately, thus constructing the comprehensive index for digital transformation [33]. Firstly, considering the rapid expansion characteristic of digital transformation, we employed the logarithmic utility function method for dimensionless processing, ensuring horizontal and vertical comparability among the indicators. The transformation formula is as follows:

$$x_{i,j,k,t}^{*}=\frac{x_{i,j,k,t}-\text{min}(x_{i,j,k,2011})}{\text{max}(x_{i,j,k,2011})-\text{min}(x_{i,j,k,2011})}\times 100$$ (1)

In the Eq (1), *i* represents the enterprise, *j* represents the primary dimension, *k* represents the secondary dimension, and *t* represents the year. *x*_*i*,*j*,*k*,*t*_ denotes the value of enterprise *i* in year *t* on dimension *j* of sub-dimension *k*. min(*x*_*i*,*j*,*k*,2011_) and max(*x*_*i*,*j*,*k*,2011_) are respectively the lower and upper thresholds of the utility function, taken as the minimum and maximum actual values of all listed enterprises in the year 2011. $x_{i,j,k,t}^{*}$ represents the value after dimensionless processing. Following this processing, the dimensionless value score range for each corresponding indicator is between 0 and 100, the higher the $x_{i,j,k,2010}^{*}$, the better the performance of enterprise *i* on the respective indicator in the year 2011. For data following the baseline year of 2011, the efficiency score of the indicator might be less than 0 or greater than 100.

After carrying out the dimensionless processing on the various dimension indicators of digital transformation, we employ the Principal Component Analysis (PCA) method to construct the index of digital transformation. Firstly, it is necessary to ascertain the weights when synthesizing various indicators. Our study determines the weights of each dimension indicator based on the coefficient of variation method and the Analytic Hierarchy Process (AHP). The results are shown in Table 3. Secondly, based on the weights assigned to each indicator, a bottom-up approach is taken for stepwise weighted summation, obtaining the variable *DT*.

**Table 3:** Weight of each dimension for the digital transformation (*DT*).

Primary Demention	Weight	Secondary Demention	Weight
Information advantage	63.91%	Corporate strategy	36.51%
		Financial accounting	26.25%
		Infrastructure	23.57%
		Business model	13.67%
Shareholder capability	36.09%	Shareholder digital background	44.75%
		Fintech	33.16%
		Internet attention	Baidu Search Index (16.25%), Guba comments (5.84%)

#### 4.2.3 Control variables

Following the studies, we select control variables including following variables [25, 38]: Company Size (*SIZE*), measured by the natural logarithm of total assets plus one. Financial Leverage (*LEVAGE*), measured by the ratio of liabilities to net assets. Return on Assets (*ROA*), measured by the ratio of net profit after tax to total assets. Company Growth (*GROWTH*), measured by the growth rate of operating income. Board Size (*BOARD*), measured by the natural logarithm of the number of board members. Independent Director Ratio (*INDEP*), measured by the ratio of independent directors to the number of board members. CEO-Chairman Duality (*DUELTY*), a dummy variable for whether the chairman and the general manager are the same person. Institutional Ownership (*FUND*), measured by the shareholding ratio of institutional investors. Shareholding Concentration (*TOP*), measured by the shareholding ratio of the largest shareholder. State Ownership (*SOE*), a dummy variable for whether it is a state-owned enterprise. Firm Age (*AGE*), measured by the natural logarithm of the number of years since the company was established. Audit Quality (*BIG4*), a dummy variable for whether the company is audited by one of the Big Four accounting firms (PwC, DTT, KPMG, EY). Newspaper Coverage (*MEDIA*), measured by the natural logarithm of the number of times a company is reported in the newspaper in a given year plus one.

### 4.3 Regression model

We establish an OLS (Ordinary Least Squares) model to estimate the impact of digital transformation:

$$RPT_{i,t}=\beta_{0}+\beta_{1}DT_{i,t-1}+\sum_{t-1}^{i}CONTROL+YEAR_{i,t}+\varepsilon_{i,t}$$ (2)

**Fig 1:**
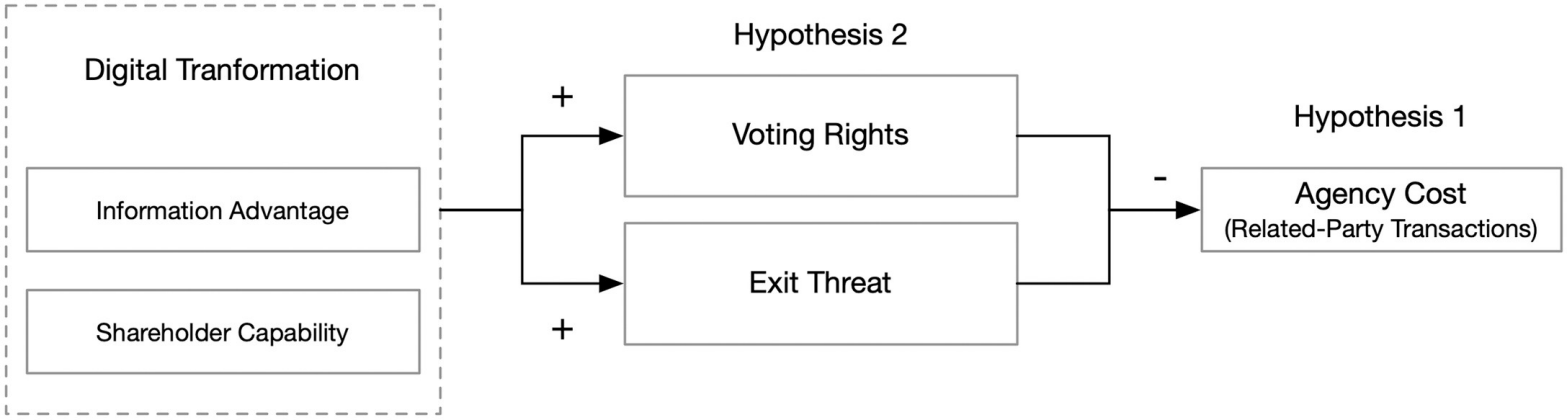
Overall hypothesis model.

In the Eq (2), *i* represents the firm, and *t* represents the year. *RPT* represents the dependent variable proxy cost in our study. digital transformation represents the core explanatory variable digital transformation. *CONTROL* represents control variables. *YEAR* and *INDUST* respectively represent the year and industry dummy variables. *β*_0_ is the intercept term, *β*_1_ is the core estimated parameter, and ε represents the random error term. In our study, we have lagged the explanatory variable and control variables by one period in the regression. To ensure the robustness of the conclusions, the t-statistics of all regression models in our study have been subjected to White heteroskedasticity-robust treatment and adjusted at the firm level for clustering [39]. Fig 1 shows the overall hypothesis framework of our research model.

## 5. Results

### 5.1 Descriptive statistics

Table 4 presents the primary descriptive statistics. The average value of RPT is 0.315, indicating that the average level of related party transactions for the sample listed companies is 31.5% of total assets, with a range of 2.316 percentage points between the maximum and minimum values. After normalization, the average value of *DT* is 0.011, with a standard deviation of 0.956. Among the *DT* sub-variables, *DT_CS* has an average of 18.917, suggesting that digital keywords are mentioned an average of 18–19 times in the annual reports of listed companies, with a maximum frequency of 277 times. *DT_FA* averages 0.306, showing that 30.6% of the sample companies utilize ERP financial management systems. *DT_INF* has an average of 0.629 and a standard deviation of 2.158, indicating significant variation in digital infrastructure investment among the companies. *DT_BM* averages 0.277, reflecting that 27.7% of the companies have adopted CRM management systems, platform economy models, or sharing economy models aided by big data technology, signifying a digital transformation in their business models. The remaining control variables align with the descriptive statistics of listed companies reported in other literature. *DT_SDB* averages 0.252, indicating that directors with IT backgrounds constitute an average of 25.2% of the board in the sample companies. *DT_FIN* has an average of 3.306 with a standard deviation of 1.142 and a maximum value of 4.514, suggesting a generally high level of fintech expertise among institutional investor shareholders of the sample companies, with notable variations. *DT_BAIDU* and *DT_GUBA* have averages of 11.293 and 9.340, respectively, close to their maximum values, indicating frequent searches and discussions by shareholders on Baidu and Guba, reflecting their strong motivation and ability to leverage digital technology for acquiring corporate information.

**Table 4:** Descriptive statistical results.

Variables	Obs.	Mean	Median	Std.	Min	Max
*RPT*	31,649	0.315	0.179	0.400	0.001	2.317
*DT*	31,649	0.011	-0.181	0.956	-1.630	2.734
*DT_CS*	31,649	18.917	3.028	43.149	0.000	277.000
*DT_FA*	31,649	0.306	0.000	0.461	0.000	1.000
*DT_INF*	31,649	0.629	0.000	2.158	0.000	19.288
*DT_BM*	31,649	0.277	0.283	0.494	0.000	1.000
*DT_SDB*	31,649	0.252	0.200	0.250	0.000	1.000
*DT_FIN*	31,649	3.306	3.791	1.142	0.157	4.514
*DT_BAIDU*	31,649	11.293	9.476	3.887	0.000	17.551
*DT_GUBA*	31,649	9.340	9.050	1.398	6.867	13.009
*TOP1*	31,649	0.342	0.321	0.148	0.090	1.000
*TOP5*	31,649	0.535	0.536	0.155	0.177	1.000
*TOP10*	31,649	0.586	0.595	0.154	0.208	1.000
*FUND*	31,649	0.380	0.385	0.236	0.000	0.871
*SIZE*	31,649	22.220	22.030	1.284	19.987	26.063
*LEVAGE*	31,649	0.423	0.414	0.206	0.059	0.890
*ROA*	31,649	0.041	0.039	0.063	-0.214	0.209
*GROWTH*	31,649	0.286	0.116	0.772	-0.400	5.360
*BOARD*	31,649	7.497	8.000	1.633	4.000	13.000
*INDEP*	31,649	0.376	0.364	0.053	0.333	0.571
*DUALTY*	31,649	0.291	0.000	0.454	0.000	1.000
*SOE*	31,649	0.336	0.000	0.472	0.000	1.000
*AGE*	31,649	18.291	18.000	5.840	6.000	33.000
*BIG4*	31,649	0.059	0.000	0.236	0.000	1.000
*MEDIA*	31,649	3.659	3.296	2.049	0.000	9.486

Note: The results in this table represent descriptive statistics of variables before applying Z-score normalization. We have included descriptive statistics for eight sub-dimensions of the digital transformation variable *DT* in the table. *DT_CS* corresponds to the Corporate Strategy dimension. *DT_FA* refers to the Financial Accounting dimension. *DT_INF* denotes the Infrastructure dimension. *DT_BM* is the Business Model dimension. *DT_SDB* stands for the Shareholder Digital Background dimension. *DT_FIN* represents the Fintech dimension. *DT_BAIDU* and *DT_GUBA* are indicators of the Internet Attention dimension.

In terms of shareholder holdings in our sample companies, *TOP1* represents the shareholding percentage of the largest shareholder, averaging 0.342, with a minimum and maximum of 0.09 and 1, respectively. This indicates that the average shareholding of the largest shareholder in the sample companies is 34.2%, with the smallest and largest shareholdings being 9% and 100%. *TOP5*, reflecting the shareholding of the top five shareholders, averages 0.535, showing that the average shareholding of the top five shareholders in the sample companies is 53.5%. *TOP10*, denoting the top ten shareholders’ holdings, averages 0.586, indicating an average shareholding of 58.6% among the top ten shareholders in the sample companies. *FUND*, which represents institutional shareholdings, averages 0.38 with a minimum and maximum of 0.001 and 0.871, respectively. This reveals that the average shareholding of institutional investor shareholders in the sample listed companies is 38%, with the smallest and largest shareholdings being 0.1% and 87.1%, indicating significant diversification in sample institutional holdings, showing a polarization trend. Overall, in our sample listed companies, the average shareholding of the largest shareholder is significantly higher than that of any single institutional investor or public shareholder. These signs indicate that the phenomena of a dominant single shareholder and oligarchic shareholding are common in Chinese listed companies. Institutional investors struggle to effectively counterbalance the largest shareholders in terms of shareholding percentages, with a generally wide gap in shareholdings [1, 3]. The largest shareholder often holds an absolute controlling position, while the combined average shareholding of institutional investors and public shareholders usually exceeds that of the largest shareholder. This phenomenon suggests that an effective balance of power between controlling shareholders and external minority shareholders exists in these companies. Investigating the changes in minority shareholder governance brought about by digital transformation in enterprises is a meaningful topic. The descriptive statistical results for the other control variables are broadly consistent with other literature on Chinese listed companies.

**Fig 2:**
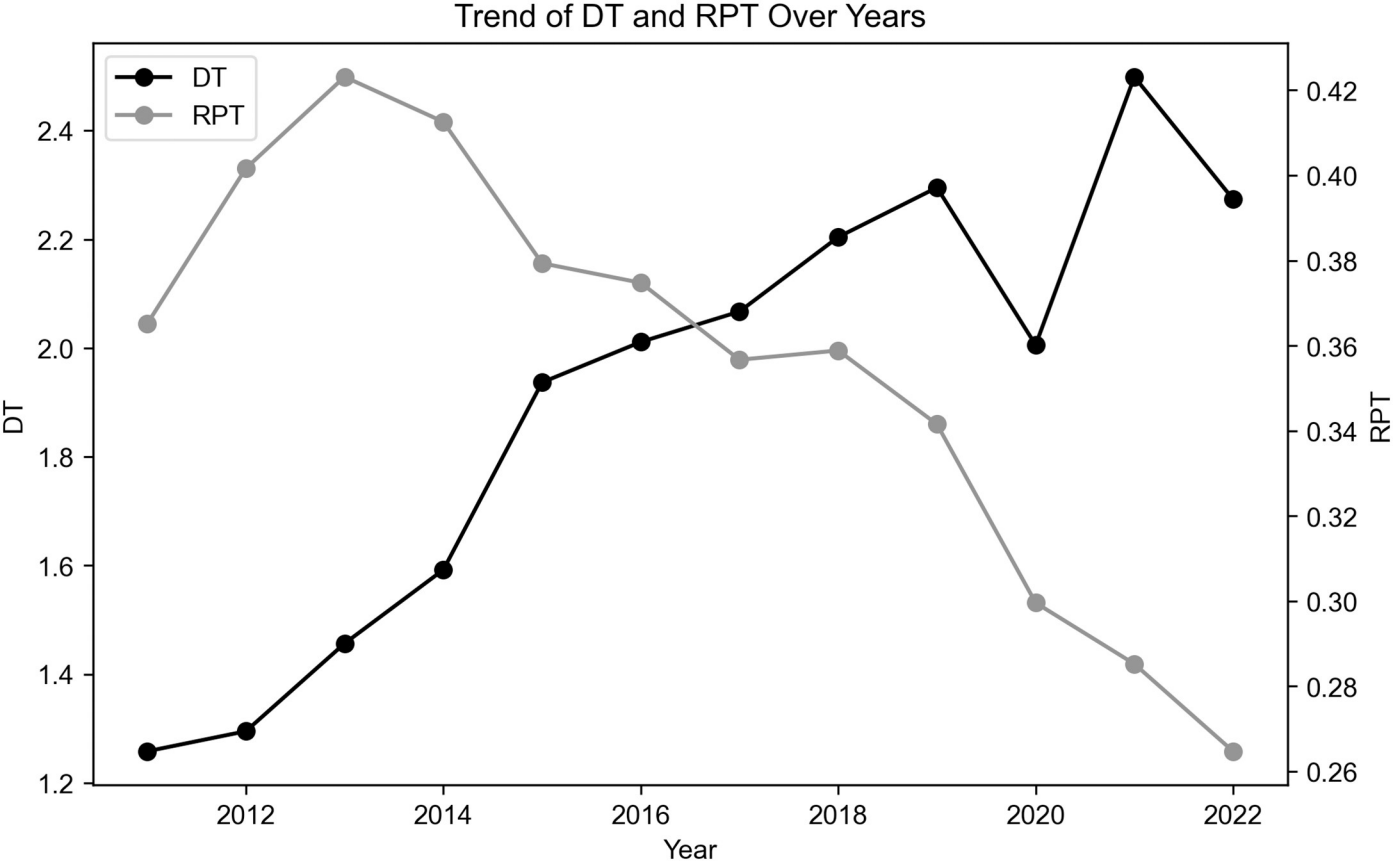
Trends in annual mean values of *DT* and *RPT*. The black line represents the average number of *DT* and shows an upward trend, and the gray line represents the number of *RPT* and shows a downward trend.

Fig 2 presents the annual mean changes of the variables *DT* and *RPT*. It is observable that the mean of *DT* gradually increases with each year, indicating an incremental yearly rise in the overall level of digital transformation among the sampled firms. Concurrently, the mean of *RPT* demonstrates a downward trend as the years progress, suggesting a decrease in the level of related-party transactions among these firms over time. These trends imply an inverse relationship between *DT* and *RPT*, to some extent, indicating a negative correlation between them.

### 5.2 The effectiveness of digital transformation variables

Our study constructs the explanatory variable *DT* for digital transformation in enterprises, based on principal component analysis. This analysis considers two aspects: the information advantage from enterprise digitalization and shareholders’ ability to interpret digital information. However, the validity of this variable in aligning with business practices requires further confirmation. We validate the effectiveness of the *DT* variable by presenting its distribution of mean, median, standard deviation, minimum, and maximum values across different industries. This is done through descriptive statistics of *DT*, categorized by industry. Following the industry classification standards of the China Securities Regulatory Commission (CSRC) for the year 2012, we conduct these descriptive statistics for *DT* at both first and second moment industry.

Table 5 presents the results of descriptive statistics for the *DT* variable, categorized by primary industry sectors as per the classification by the China Securities Regulatory Commission. The results show that companies with higher average *DT* values are concentrated in industries like Information Technology, Finance and Insurance, Arts, Entertainment, and Recreation, Professional, Scientific, and Technical Services, and Health Care and Social Assistance. This aligns with the tertiary sector’s reliance on digital technology, platforms, and customer engagement. Notably, the Information Technology sector has an average *DT* of 3.287, significantly higher than other industries, indicating its advanced digital transformation due to its core reliance on digital technology. Conversely, lower *DT* averages are observed in resource-intensive industries such as Agriculture, Forestry, Fishing and Hunting, Accommodation and Food Services, Utilities, Mining, Quarrying, and Oil and Gas Extraction, where resource advantages play a more prominent role than digital transformation. These results, consistent with real-world business practices and general perception, affirm the validity of the *DT* variable in reflecting digital transformation across different industries.

**Table 5:** Descriptive statistics of *DT* by first-moment industry.

Industry	Obs.	Mean	Median	Std.	Min	Max
Information Technology	2,164	1.372	1.588	0.647	-1.487	2.734
Finance and Insurance	302	0.84	1.01	0.86	-1.399	1.952
Other Services	4	0.591	0.704	0.996	-0.524	1.479
Real Estate and Rental and Leasing; Professional, Scientific, and Technical Services	80	0.375	0.394	0.908	-1.229	1.838
Health Care and Social Assistance	413	0.371	0.416	0.896	-1.532	2.366
Arts, Entertainment, and Recreation	44	0.318	0.409	0.914	-1.341	1.791
Educational Services	435	0.31	0.38	0.81	-1.349	1.919
Professional, Scientific, and Technical Services	376	0.271	0.34	0.91	-1.345	2.321
Manufacturing	20,332	0.031	-0.105	0.903	-1.536	2.71
Wholesale and Retail Trade	1,588	-0.067	-0.307	0.9	-1.516	2.033
Transportation and Warehousing	832	-0.176	-0.35	0.796	-1.52	1.876
Construction	904	-0.189	-0.384	0.793	-1.505	2.137
Conglomerates	101	-0.344	-0.472	0.672	-1.492	1.347
Accommodation and Food Services	210	-0.345	-0.45	0.7	-1.49	2.007
Real Estate, Rental and Leasing	1,434	-0.441	-0.631	0.681	-1.53	2.327
Utilities	1,244	-0.468	-0.631	0.702	-1.532	2.025
Agriculture, Forestry, Fishing and Hunting	420	-0.498	-0.688	0.714	-1.485	2.23
Mining, Quarrying, and Oil and Gas Extraction	718	-0.546	-0.696	0.626	-1.485	1.6
Others	2,135	-0.906	-1.035	0.608	-1.635	2.049

Table 6 displays the descriptive statistical results of the *DT* variable categorized by secondary industries (sub-sectors) as defined by the China Securities Regulatory Commission. As seen in Table 5, industries with higher *DT* averages are mainly in high-tech sectors like Software and Computer Services, Internet Services and Infrastructure, Telecommunications, and financial services such as Postal Services, Banking Services, Insurance, and Capital Markets. These industries are characterized by a higher degree of digital transformation. Conversely, lower *DT* averages are found in sectors heavily reliant on resources, such as Agriculture Operations, Agribusiness, Metals and Mining, Railroads, and Forestry and Logging, where digital transformation is less pronounced. Overall, the results in Table 5, consistent with real-world business practices, not only complement those in Table 4 but also further validate the construction and effectiveness of the *DT* variable.

**Table 6:** Descriptive statistics of *DT* by second-moment industry.

Industry	Obs.	Mean	Median	Std.	Min	Max
Software and Information Technology Services	1,622	1.479	1.627	0.555	-1.224	2.734
Postal Services	24	1.325	1.487	0.545	-0.299	2.019
Monetary Financial Services	95	1.231	1.603	0.733	-1.074	1.952
Internet and Related Services	398	1.057	1.174	0.774	-1.24	2.616
Telecommunications, Broadcast Television, and Satellite Transmission Services	144	1.039	1.342	0.808	-1.487	2.368
Instrument and Apparatus Manufacturing	373	0.93	1.043	0.763	-1.265	2.463
Computer, Communication, and Other Electronic Equipment Manufacturing	2,949	0.758	0.885	0.822	-1.489	2.71
Capital Market Services	139	0.73	0.961	0.806	-1.399	1.903
Furniture Manufacturing	154	0.686	0.852	0.818	-1.188	2.58
Repair of Motor Vehicles, Electronics, and Consumer Goods	4	0.591	0.704	0.996	-0.524	1.479
Insurance Industry	16	0.553	0.822	0.934	-1.121	1.934
Culture and Arts	49	0.532	0.57	0.953	-1.349	1.894
Other Financial Services	52	0.51	0.765	0.952	-1.173	1.947
News and Publishing	202	0.5	0.662	0.755	-1.165	1.833
Technology Promotion and Application Services	6	0.438	0.447	0.348	0.074	1.009
Business Services	387	0.42	0.472	0.885	-1.532	2.366
Electrical Machinery and Equipment Manufacturing	2,028	0.376	0.436	0.846	-1.488	2.553
Health Services	80	0.375	0.394	0.908	-1.229	1.838
Building Installation	11	0.358	0.178	1.096	-1.13	1.876
Loading, Unloading, Handling, and Transportation Agency Services	43	0.353	0.428	0.765	-1.227	1.789
Professional Technical Services	313	0.332	0.408	0.917	-1.28	2.321
Education	44	0.318	0.409	0.914	-1.341	1.791
Warehousing	73	0.313	0.436	0.943	-1.49	1.803
Sports	6	0.285	0.351	0.846	-0.928	1.448
Specialized Equipment Manufacturing	1,895	0.25	0.329	0.874	-1.516	2.326
Building Decoration and Other Construction Industry	230	0.172	0.168	0.826	-1.309	1.815
Manufacture of Cultural, Educational, and Sports Goods	129	0.166	0.166	0.829	-1.314	1.89
Printing and Media Reproduction	98	0.13	-0.144	0.925	-1.21	1.997
General Equipment Manufacturing	1,178	0.086	0.041	0.902	-1.528	2.243
Textile Apparel and Accessories	339	0.045	-0.064	0.81	-1.481	2.329
Automobile Manufacturing	1,055	0.036	0.051	0.789	-1.52	1.936
Broadcasting, Television, Film Production, and Recording	178	0.033	-0.139	0.751	-1.272	1.919
Residential Building Construction	14	-0.038	-0.478	0.825	-0.804	1.574
Manufacturing of Transport Equipment for Railways, Ships, Aerospace, and Others	444	-0.043	-0.202	0.811	-1.474	2.068
Metal Products Industry	567	-0.061	-0.149	0.857	-1.526	2.35
Retail Trade	863	-0.064	-0.274	0.865	-1.515	2.033
Wholesale Trade	725	-0.072	-0.36	0.941	-1.516	1.912
Research and Experimental Development	57	-0.083	-0.234	0.838	-1.345	1.55
Wood Processing and Manufacture of Wood, Bamboo, Rattan, Palm, and Straw Products	84	-0.094	-0.273	0.901	-1.365	1.71
Ecological Protection and Environmental Governance	271	-0.163	-0.285	0.774	-1.474	1.662
Comprehensive Utilization of Waste Resources	45	-0.173	-0.212	0.71	-1.204	1.099
Road Transportation	326	-0.187	-0.369	0.715	-1.505	1.822
Rubber and Plastic Products	655	-0.191	-0.337	0.754	-1.502	1.774
Mining Support Activities	127	-0.193	-0.332	0.751	-1.457	1.6
Food Manufacturing	402	-0.225	-0.419	0.772	-1.414	1.892
Paper and Paper Products	283	-0.244	-0.478	0.822	-1.502	2.542
Leather, Fur, Feather and Related Products and Footwear Manufacturing	85	-0.259	-0.423	0.801	-1.341	1.777
Other Manufacturing	172	-0.261	-0.408	0.732	-1.361	1.493
Air Transport	115	-0.273	-0.391	0.681	-1.473	1.495
Manufacturing of Beverages, Tea, and Refined Tea	404	-0.295	-0.46	0.753	-1.456	1.778
Accommodation	67	-0.317	-0.424	0.706	-1.492	1.347
Animal Husbandry	133	-0.319	-0.601	0.815	-1.466	2.23
Civil Engineering Construction	577	-0.328	-0.525	0.728	-1.52	1.69
Non-metallic Mineral Products	809	-0.339	-0.474	0.724	-1.524	1.879
Conglomerates	210	-0.345	-0.45	0.7	-1.49	2.007
Processing of Agricultural and Sideline Food Products	426	-0.348	-0.58	0.722	-1.514	1.811
Rental Services	26	-0.348	-0.637	0.756	-1.168	1.574
Textile Industry	400	-0.348	-0.579	0.773	-1.519	1.766
Chemical Fiber Manufacturing	235	-0.388	-0.614	0.785	-1.496	1.752
Non-metallic Mineral Mining and Quarrying	7	-0.39	-0.603	0.515	-1.137	0.251
Catering Services	34	-0.396	-0.522	0.606	-1.333	0.899
Smelting and Rolling of Ferrous Metals	309	-0.413	-0.618	0.737	-1.512	1.865
Water Transport	283	-0.417	-0.61	0.686	-1.491	2.137
Pharmaceutical Manufacturing	1,870	-0.429	-0.627	0.685	-1.532	1.829
Chemical Raw Materials and Chemical Products Manufacturing	2,155	-0.449	-0.652	0.726	-1.536	1.758
Gas Production and Supply	175	-0.456	-0.632	0.584	-1.366	0.883
Smelting and Rolling of Non-ferrous Metals	624	-0.462	-0.637	0.644	-1.519	1.586
Real Estate	1,244	-0.468	-0.631	0.702	-1.532	2.025
Water Production and Supply	143	-0.468	-0.594	0.684	-1.501	1.727
Agriculture	148	-0.514	-0.683	0.655	-1.456	1.571
Oil and Natural Gas Extraction	52	-0.515	-0.59	0.531	-1.411	0.613
Electric Power and Heat Production and Supply	680	-0.519	-0.662	0.624	-1.53	1.829
Public Facilities Management	165	-0.54	-0.825	0.72	-1.474	2.327
Fishing	84	-0.546	-0.82	0.709	-1.485	1.063
Agriculture, Forestry, Animal Husbandry, and Fishery Services	11	-0.55	-0.889	0.727	-1.225	1.216
Coal Mining and Washing	255	-0.577	-0.769	0.613	-1.485	1.508
Non-ferrous Metal Mining and Dressing	218	-0.656	-0.723	0.53	-1.437	1.456
Oil Processing, Coking, and Nuclear Fuel Processing	165	-0.672	-0.864	0.592	-1.474	1.394
Railway Transport	40	-0.767	-0.882	0.385	-1.234	0.253
Ferrous Metal Mining and Dressing	59	-0.81	-0.897	0.5	-1.459	0.924
Forestry	44	-0.881	-0.939	0.349	-1.438	0.228
Others	2,135	-0.906	-1.035	0.608	-1.635	2.049

### 5.3 Baseline regression: The main effect of digital transformation

Table 7 reports the regression results for Eq (2). Columns (1) and (2) represent the regression results without and with control variables, respectively. The results show that, the coefficient in front of the main explanatory variable *DT* is -0.096(p<0.01), significantly negative at the 1% level. This indicates that for each standard deviation increase in the enterprise digitalization variable *DT*, the related transaction variable *RPT* will decrease by 9.6 percentage points. After incorporating control variables, the coefficient between *RPT* and *DT* is -0.039(p<0.01), also significantly negative at the 1% level. This indicates that for each standard deviation increase in the enterprise digitalization variable *DT*, the related transaction variable *RPT* will decrease by 3.9 percentage points. These results show a significant correlation between digital transformation and related transactions, which indicates that as the level of digital transformation increases, the proportion of related-party transaction amounts to total assets decreases. The decline in the level of related-party transactions makes it more difficult for controlling shareholders to seek private benefits through related-party transactions, thus reducing the agency costs caused by the conflicts of interest between controlling shareholders and minority shareholders. Therefore, digital transformation improves the governance effects of minority shareholders by reducing agency costs, protecting the interests of minority shareholders, thus supporting Hypothesis 1.

**Table 7:** The main effect of digital transformation on agency cost. All standard errors have been processed using White’s heteroskedasticity robust treatment and are adjusted for clustering at the firm level.

Variables	(1)	(2)
	*RPT*	*RPT*
*DT*	-0.096*** (-8.13)	-0.039*** (-3.81)
*SIZE*		-0.094*** (-5.83)
*LEVAGE*		0.329*** (24.01)
*ROA*		-0.067*** (-7.23)
*GROWTH*		0.039*** (5.80)
*BOARD*		-0.017 (-1.41)
*INDEP*		-0.017 (-1.50)
*DUALTY*		-0.043** (-2.34)
*TOP*		0.039*** (3.54)
*FUND*		0.022** (2.08)
*SOE*		-0.008 (-0.28)
*AGE*		0.063*** (5.24)
*BIG4*		-0.153*** (-2.93)
*MEDIA*		-0.031** (-2.37)
Firm FE	YES	YES
Year	YES	YES
N	31,649	31,649
Adj-R^2^	0.014	0.142

Note:

*, ** and *** denote statistical significance at the 10%, 5%, 1% level. T-statistics are in parentheses. In the regression process of all our models, we clustered at the firm level to control for firm FE effects.

### 5.4 Mediation mechanism investigation

#### 5.4.1 Minority shareholders’ voting rights at shareholders’ meetings

Minority shareholders can participate in corporate decision-making and enhance their influence on the governance of listed enterprises by attending shareholders’ meetings and actively exercising their voting rights [28]. Therefore, we use *ATTEND*, which is the attendance rate of minority shareholders in shareholders’ meetings, to measure the extent to which minority shareholders exercise their voting rights. The calculation method is the ratio of the number of minority shareholders (excluding the top 5 shareholders of the listed company) attending the annual shareholders’ meeting to the total number of shareholders attending the annual shareholders’ meeting. Based on this, we adopt the stepwise mediation effect test, as proposed by *Baron and Kenny (1986)*, to analyze the relationship among digital transformation, the attendance rate of minority shareholders, and agency costs [40]. Our stepwise mediation effect test model is represented in Eqs (3) and (4).


$$ATTEND_{i,t}=\beta_{0}+\beta_{1}DT_{i,t-1}+\sum_{t-1}^{i}CONTROL+YEAR_{i,t}+\varepsilon_{i,t}$$ (3)

$$RPT_{i,t}=\beta_{0}+\beta_{1}ATTEND_{i,t-1}+\sum_{t-1}^{i}CONTROL+YEAR_{i,t}+\varepsilon_{i,t}$$ (4)

Eq (3) illustrates the impact of the independent variable *DT* on the mediating variable *ATTEND*, while Eq (4) illustrates the effect of the mediating variable *ATTEND* on the dependent variable *RPT*. If the coefficient *β*_1_ in Eq (3) is statistically significant and positive, it indicates a significant positive correlation between *DT* and *ATTEND*, suggesting that corporate digital transformation significantly increases the attendance rate of minority shareholders. Under this premise, if the coefficient *β*_1_ in Eq (4) is statistically significant and negative, it signifies a significant negative relationship between *ATTEND* and *RPT*, indicating a mediating effect. This means that corporate digital transformation can reduce the agency costs associated with related transactions by increasing the attendance rate of minority shareholders, therefore Hypothesis 2 is supported.

Column (1) of Table 8 presents the regression analysis result for Eq (3). The results show a coefficient of 0.063 between *DT* and *ATTEND*, which is significantly positive at the 1% level. This finding implies that one standard deviation increase in *DT* leads to a 6.3% rise in *ATTEND*. It suggests a positive relationship between the extent of digital transformation and the participation of minority shareholders in annual general meetings. Digital transformation appears to bolster the willingness of minority shareholders to participate in such meetings and to exercise their voting rights. In Column (2) of Table 8, the regression results for Eq (4) indicate that the coefficient between *ATTEND* and *RPT* is -0.042, significantly negative at the 1% level. This outcome indicates that an increase in the attendance rate of minority shareholders at the annual general meetings significantly reduces the level of related party transactions within the company. Specifically, one standard deviation increases in *ATTEND* results in a 4.2% decrease in *RPT*.

**Table 8:** The mechanism of digital transformation on agency cost. All standard errors have been processed using White’s heteroskedasticity robust treatment and are adjusted for clustering at the firm level.

Variables	(1)	(2)	(3)	(4)
	*ATTEND*	*RPT*	*EXIT*	*RPT*
*DT*	0.063*** (6.82)		0.031*** (4.39)	
*ATTEND*		-0.042*** (-3.38)		
*EXIT*				-0.016** (-2.34)
Control Variables	YES	YES	YES	YES
Firm FE	YES	YES	YES	YES
Year	YES	YES	YES	YES
Observations	31,649	31,649	31,649	31,649
Adj-R^2^	0.183	0.142	0.348	0.142

Note:

*, ** and *** denote statistical significance at the 10%, 5%, 1% level. T-statistics are in parentheses. The control variables are the same as those in Table 4. Due to space constraints, it is not shown here.

In summary, by constructing and testing a stepwise mediation effect model, we have successfully validated the mechanism of minority shareholders’ attendance rate. Specifically, enterprise digitalization can reduce agency costs by increasing the attendance of minority shareholders at shareholders’ meetings. This finding is consistent with Hypothesis H2.

#### 5.4.2 Minority shareholders’ exit threat

The exit threat of minority shareholders refers to the scenario where minority shareholders, facing unjust decisions by controlling shareholders that infringe on their legitimate interests, reduce their shareholding ratio or exit the shareholder ranks by massively selling the shares of the listed company, thereby constraining the controlling shareholders from encroaching on the interests of minority shareholders. Following the study by *Dou et al*. *(2018)*, we employ the variable *EXIT* to measure the extent of minority shareholders’ exit threat [41]. The calculation formula for *EXIT* is as follows:

$$EXIT_{i,t}=LIQUID_{i,t}\times COMP_{i,t}$$ (5)

$$COMP_{i,t}=-1\times \sum_{k=1}^{5}(BLOCK_{i,k,t}/BLOCK_{i,t}{)}^{2}$$ (6)

In these equations, *i* represents the firm, and *t* represents the year. In the Eq (5), *LIQUID* represents the annual average of the daily turnover rate of the listed company’s circulating shares, while *COMP* represents the level of competition between large shareholders and minority shareholders. In Eq (6), *BLOCK*_*i*,*k*,*t*_ represents the shareholding ratio of the *k*^*th*^ large shareholder in company *i* during year *t*. The right part of Eq (6) represents the sum of the shareholding ratios of the top five large shareholders in company *i* during year *t*. The larger the sum is, the higher the concentration of equity in the company, and the lower the competition between controlling shareholders and external minority shareholders. This is because a higher concentration of equity leads to diminished constraints on the decisions of major shareholders from minority shareholders. The capital amount represented by minority shareholder stakes becomes less significant to major shareholders. Consequently, the threat of minority shareholders exiting is lower. After reciprocal transformation, the larger the value of *COMP*, the higher the level of competition between controlling shareholders and external minority shareholders. Therefore, *EXIT* represents the synergistic effect of the liquidity of the listed company’s shares and the competition between large shareholders and minority shareholders. The stronger the liquidity of the listed company’s shares, and the higher the level of competition between large shareholders and minority shareholders. Therefore, the larger the value of *EXIT*, indicating a stronger exit threat faced by the listed company from minority shareholders.

Following the same approach as the previous section, we constructed stepwise mediation effect test models Eqs (7) and (8) to validate the mechanism of the exit threat posed by minority shareholders:

$$EXIT_{i,t}=\beta_{0}+\beta_{1}DT_{i,t-1}+\sum_{t-1}^{i}CONTROL+YEAR_{i,t}+\varepsilon_{i,t}$$ (7)

$$RPT_{i,t}=\beta_{0}+\beta_{1}EXIT_{i,t-1}+\sum_{t-1}^{i}CONTROL+YEAR_{i,t}+\varepsilon_{i,t}$$ (8)

In these equations, Eq (7) illustrates the impact of the independent variable *DT* on the mediating variable *EXIT*, while Eq (8) illustrates the effect of the mediating variable *EXIT* on the dependent variable *RPT*. If the coefficient *β*_1_ in Eq (7) is statistically significant and positive, it indicates a significant positive correlation between *DT* and *EXIT*, suggesting that corporate digital transformation significantly increases the exit threat of minority shareholders. Under this premise, if the coefficient *β*_1_ in Eq (8) is statistically significant and negative, it signifies a significant negative relationship between *EXIT* and *RPT*, indicating a mediating effect. This means that corporate digital transformation can reduce the agency costs associated with related transactions by increasing the exit threat of minority shareholders, thereby supporting Hypothesis 2 of our study.

Column (3) of Table 8 reports the regression results of Eq (7). We can see that the coefficient between *DT* and *EXIT* is 0.031, significantly positive at the 1% level. This indicates that for one standard deviation increase in the enterprise digitalization variable *DT*, the exit threat variable for minority shareholders, *EXIT*, will increase by 3.1%. Such findings signify a positive correlation between digital transformation and the exit threat among minority shareholders. Additionally, digital transformation amplifies the potential for minority shareholders to impose constraints on major shareholders via exit threats. Column (4) of Table 8 reports the regression results of Eq (8). We observe that the coefficient between *EXIT* and *RPT* is -0.016, significant at the 5% level in a negative direction. This outcome suggests that the exit threat posed by minority shareholders is significantly negatively correlated with the level of related transactions within a company. Specifically, for one standard deviation increase in *EXIT*, *RPT* decreases by 1.6%.

In summary, by constructing and testing a stepwise mediation effect model, we have successfully validated the mechanism of minority shareholders’ exit threat. Specifically, corporate digitalization can reduce agency costs by increasing the exit threat of minority shareholders. This finding is in alignment with Hypothesis H2.

### 5.5. Robustness test

#### 5.5.1 Endogeneity problem: Self-selection bias

Considering the characteristics of enterprises, firms engaged in digital transformation are likely to differ from those not engaged in digital transformation. This distinction may lead to pre-existing differences in the degree and effectiveness of minority shareholder involvement in corporate governance between digitally transforming and non-digitally transforming firms. Therefore, our study might face an endogeneity issue due to variable self-selection bias. To mitigate this issue, our study employs the method proposed by *Rosenbaum and Rubin (1985)*, utilizing the Propensity Score Matching (PSM) method to match the treatment group samples with control group samples based on a one-to-multiple nearest neighbor matching principle, and conducts regressions on the matched samples [42]. Hence, we define the explained variable *DT_D*, for the degree of digital transformation of firms, split according to the annual median. If a sample firm’s *DT* value in a particular year exceeds the annual median for *DT*, *DT_D* is set to 1 (treatment group). Otherwise, it is set to 0 (control group). Then, we perform matching using the control variables from the basic regression model as covariates. Table 9 reports the balance test results after PSM matching, showing that there is no significant difference in the means of the covariates between the treatment and control groups post-matching.

**Table 9:** The balance test results after PSM matching.

Variables	Before Matching			After Matching		
	Mean (Treatment)	Mean (Control)	%Bias	Mean (Treatment)	Mean (Control)	% Bias
*SIZE*	0.031	-0.039	7.000	-0.027	-0.023	-0.400
*LEVAGE*	-0.064	0.084	-14.700	0.019	-0.028	4.600
*ROA*	0.011	-0.015	2.600	0.005	0.025	-2.000
*GROWTH*	0.001	-0.001	0.100	0.010	0.013	-0.300
*BOARD*	-0.078	0.102	-18.100	0.038	-0.001	3.900
*INDEP*	0.049	-0.064	11.300	-0.030	-0.008	-2.200
*DUALTY*	0.336	0.248	19.300	0.269	0.289	-4.400
*TOP*	-0.091	0.118	-21.000	0.054	0.001	5.300
*FUND*	-0.055	0.072	-12.800	0.026	-0.017	4.300
*SOE*	0.261	0.408	-31.500	0.350	0.309	8.700
*AGE*	0.105	-0.134	24.000	-0.057	0.003	-6.000
*BIG4*	0.061	0.058	1.200	0.060	0.058	0.800
*MEDIA*	0.051	0.094	-4.300	0.095	0.085	1.000

Note: The results in this table represent the balance test results after PSM matching after applying Z-score normalization to all control variables.

**Fig 3:**
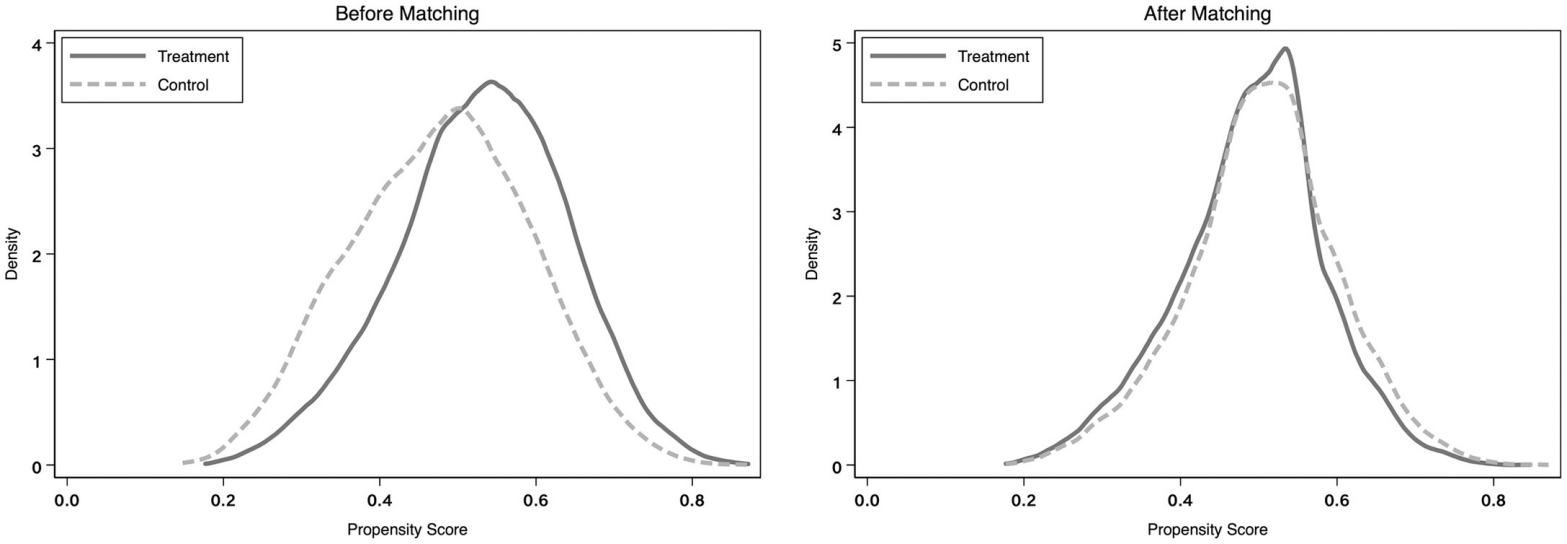
Kernel density function graph of samples before and after PSM.

Fig 3 displays the Kernel Density Function graphs of samples before and after matching through the Propensity Score Matching (PSM) method. It is evident that after the application of the PSM method for 1-to-M nearest neighbor matching based on feature variables, the density functions of the treatment group samples (companies with a higher degree of digital transformation) and control group samples (companies with a lower degree of digital transformation) tend to converge. Furthermore, there are no significant differences in the values of the feature variables.

After eliminating 4,899 unmatched samples, we obtained 13,199 treatment group samples and 13,551 control group samples, totaling 26,750 firm-year samples. Table 10 reports the regression results after PSM matching. When *RPT* is the dependent variable, the coefficient between *DT* and *RPT* is significantly negative. When *ATTEND* and *EXIT* are the dependent variables, the coefficient between *DT* and *ATTEND* and the coefficient between *DT* and *EXIT* are significantly positive, consistent with the main hypotheses of our study, indicating that the results of our study are still robust.

**Table 10:** Regression with balanced samples after PSM. All standard errors have been processed using White’s heteroskedasticity robust treatment and are adjusted for clustering at the firm level.

Variables	(1)	(2)	(3)
	*RPT*	*ATTEND*	*EXIT*
*DT*	-0.037*** (-3.14)	0.077*** (7.94)	0.036*** (4.12)
Control Variables	YES	YES	YES
Firm FE	YES	YES	YES
Year	YES	YES	YES
Observations	26,750	26,750	26,750
Adj-R^2^	0.133	0.378	0.362

Note:

*, ** and *** denote statistical significance at the 10%, 5%, 1% level. T-statistics are in parentheses. The control variables are the same as those in Table 4. Due to space constraints, it is not shown here.

#### 5.5.2 Endogeneity problem: Sample self-selection

Given that the construction of our *DT* variable mainly considers the dimensions of information advantage and shareholder capability, there might be an oversight of other crucial factors affecting the level of digital transformation in firms. Therefore, this study might encompass endogeneity issues due to sample selection bias. To address this issue, we construct an instrumental variable (IV) to substitute the original explanatory variable *DT*, and re-run the regression using the *Heckman (1979)* two-stage method [43].

Following the approach of *Huang et al*. *(2019)*, we use the historical postal and telecommunications data from 1984 of the cities where the companies are located as an instrumental variable for corporate digital transformation [44]. We chose this instrumental variable for two main reasons. First, the internet, as an extension of traditional communication technologies, is influenced by local historical telecom infrastructure in terms of technology level and usage habits, meeting the instrument’s exclusivity requirement. Second, the impact of traditional telecom tools like landline phones on economic development has diminished with declining usage, satisfying the instrument’s relevance. Since the original data of this instrument are cross-sectional, they cannot be directly applied to panel data econometric analysis. To address this, we follow the approach of *Nunn and Qian (2014)* and introduce a time-varying variable to construct a panel instrumental variable [45]. Specifically, we create an interaction term between the number of internet users in a company’s location from the previous year and the number of telephones per 10,000 people in 1984 in the same location. We name this variable *TELECOM*, using it as an instrumental variable for the digital transformation level of the companies in that year.

Table 11 presents the results of the Heckman two-stage regression using the instrumental variable. Initially, in the first stage of the regression, the variable *DT_D* is regressed against *TELECOM* using Probit regression. The coefficient between *DT_D* and *TELECOM* is significantly positive at the 1% level. After conducting a Durbin-Wu-Hausman (DWH) test on the endogenous variable *DT_D*, the Cragg-Donald F statistic and the Kleibergen-Paap rk F statistic are 31.92 and 31.88, respectively, significantly exceeding the critical value of 16.38 for the weak instrument variable test at the 10% level, indicating that the selection of the instrumental variable meets the relevance assumption. Subsequently, based on the first-stage regression results, the inverse Mills ratio (IMR) is calculated. The IMR is then incorporated into the second-stage regression model. The coefficients between the dependent variables and *DT_D* in the second-stage regression results is consistent with the signs observed in previous regression results and are significant, further demonstrating the robustness of our study’s findings.

**Table 11:** Heckman two-stage regression approach. All standard errors have been processed using White’s heteroskedasticity robust treatment and are adjusted for clustering at the firm level.

Variables	First Stage	Second Stage		
	*DT_D*	*RPT*	*ATTEND*	*EXIT*
*TELECOM*	0.050*** (5.64)			
*DT_D*		-0.040*** (-3.60)	0.059*** (5.88)	0.035*** (4.48)
*IMR*		-0.095 (-1.03)	-0.083*** (-2.81)	-0.021** (-2.41)
Control Variables	YES	YES	YES	YES
Firm FE	YES	YES	YES	YES
Year	YES	YES	YES	YES
Observations	31,610	31,610	31,610	31,610
Adj-R^2^	0.139	0.144	0.380	0.367
Cragg-Donald F	31.92			
Kleibergen-Paap rk F	31.88			

Note:

*, ** and *** denote statistical significance at the 10%, 5%, 1% level. T-statistics are in parentheses. The control variables are the same as those in Table 4. Due to space constraints, it is not shown here.

#### 5.5.3 Endogeneity problem: Exogenous shock test

We acknowledge that the extent of digital transformation often depends on the level of marketization, the development of the digital economy, and the degree of openness in the company’s location. These factors also profoundly influence the business development model and internal governance of a company. Thus, to robustly assess whether digital transformation effectively enhances the governance efficiency of minority shareholders and affects their attendance at general meetings and exit threats, it is necessary to conduct an exogenous shock test. Drawing on the study by *Zhao et al*. *(2022)*, we use the network infrastructure upgrade under the “Broadband China” policy pilot as an exogenous policy shock and employ a Difference-in-Differences (DID) approach to evaluate this issue [46]. On one hand, the deep transformation of corporate digitalization is inseparable from the support of network infrastructure, where improvements in network performance and service quality depend on these upgrades. On the other hand, the expansive nature of the pilot policy provides a quasi-natural experimental research strategy.

In August 2013, the State Council of China, guided by the “National Informatization Development Strategy 2006–2020”, issued the “Notification on the ‘Broadband China’ Strategy and Implementation Plan”. According to this, the Chinese government has been gradually advancing the construction of broadband and other network infrastructures in phases. By now, the Ministry of Industry and Information Technology and the National Development and Reform Commission have selected 120 cities (groups) in three batches in 2014, 2015, and 2016 as “Broadband China” demonstration points. After being selected, these cities (groups) focused on expanding broadband user scale, speeding up broadband networks, and increasing network coverage to serve economic and social development. After a construction period of about three years, the selected cities have achieved national leadership in broadband access capability and broadband user penetration rate. Consequently, companies in “Broadband China” demonstration cities might have a higher degree of digital transformation. Based on this, we construct a multi-period DID model to test the impact of the “Broadband China” exogenous shock event on minority shareholder governance, as shown in Eq (9).


$$RPT/ATTEND/EXIT_{i,t}=\beta_{0}+\beta_{1}TreatPost_{i,t-1}+\sum_{t-1}^{i}CONTROL+YEAR_{i,t}+\varepsilon_{i,t}$$ (9)

**Fig 4:**
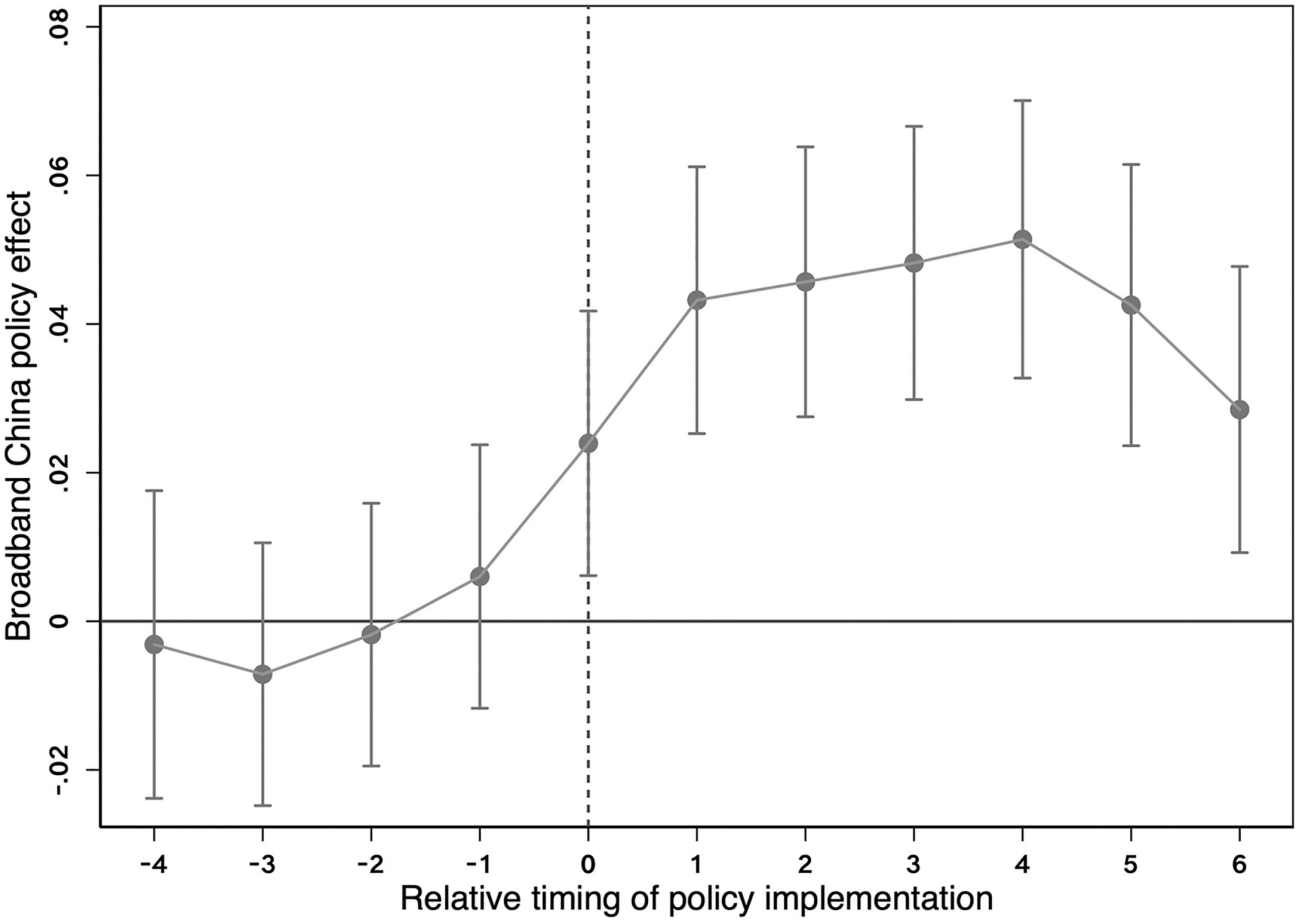
Parallel trends assumption test.

In the Eq (9), *TreatPost* indicates whether a sample company’s city is part of the “Broadband China” pilot. It takes a value of 1 if included and 0 otherwise. The definitions of other variables remain consistent with those in our previous models. Table 12 reports the results of the exogenous shock test. The regression coefficient of *TreatPost* aligns in sign with the baseline regression and mechanism test results, and is statistically significant. This suggests that the “Broadband China” policy pilot can lower the level of related party transactions by increasing minority shareholders’ attendance at shareholders’ meetings and their threat of exit, indicating enhanced governance efficiency among minority shareholders. Additionally, as shown in Fig 4, before the implementation of the “Broadband China” policy pilot, there were no significant differences in the regression coefficients between the treatment and control groups, meeting the parallel trends assumption. The test of the “Broadband China” exogenous shock event supports the conclusion that digital transformation in enterprises can improve the governance efficiency of minority shareholders.

**Table 12:** Exogenous shock test of “Broadband China” event. All standard errors have been processed using White’s heteroskedasticity robust treatment and are adjusted for clustering at the firm level.

Variables	(1)	(2)	(3)
	*RPT*	*ATTEND*	*EXIT*
*TreatPost*	-0.048* (-1.85)	0.060*** (2.86)	0.042** (2.49)
Control Variables	YES	YES	YES
Firm FE	YES	YES	YES
Year	YES	YES	YES
Observations	28,492	28,492	28,492
Adj-R^2^	0.143	0.368	0.367

Note:

*, ** and *** denote statistical significance at the 10%, 5%, 1% level. T-statistics are in parentheses. The control variables are the same as those in Table 4. Due to space constraints, it is not shown here.

#### 5.5.4 Exclude information technology industry from the sample

Due to the close correlation between core competitiveness and the level of digitalization in high-tech enterprises engaged in information technology, there is a natural connection with digitalization. These enterprises have extensively utilized digital technology since their inception or even during their establishment phase, resulting in a lower marginal utility from subsequent digital transformation. Therefore, compared to non-tech enterprises, the impact of digital transformation on minority shareholder governance might be lesser in these tech enterprises engaged in information technology [5]. To test whether digital transformation affects corporate governance in non-information technology enterprises, our study excludes information technology enterprises from the sample and performs the regression analysis again. We define the tech industry according to the China Securities Regulatory Commission’s 2012 industry classification, which includes computer, communication, and other electronic equipment manufacturing (Industry code C39), as well as information transmission, software, and information technology services (Industry codes I63, I64, I65). The regression results after excluding the information technology industry, as shown in Table 13, are consistent with the basic regression results, demonstrating robustness.

**Table 13:** Regression after excluding information technology industry. All standard errors have been processed using White’s heteroskedasticity robust treatment and are adjusted for clustering at the firm level.

Variables	(1)	(2)	(3)
	*RPT*	*ATTEND*	*EXIT*
*DT*	-0.035*** (-2.75)	0.110*** (10.28)	0.018** (2.09)
Control Variables	YES	YES	YES
Firm FE	YES	YES	YES
Year	YES	YES	YES
Observations	26,514	26,514	26,514
Adj-R^2^	0.131	0.383	0.349

Note:

*, ** and *** denote statistical significance at the 10%, 5%, 1% level. T-statistics are in parentheses. The control variables are the same as those in Table 4. Due to space constraints, it is not shown here.

#### 5.5.5 Exclude COVID-19 period from the sample

During the COVID-19 pandemic, many Chinese enterprises faced difficulties in conducting offline production and operations due to the spread of the virus and related prevention and control measures. In fact, the outbreak of COVID-19 may have forced many offline businesses to shift their operations online and engage in digital transformation activities. Consequently, including the pandemic period in our sample could potentially affect our research results. To address this, we excluded the period of the COVID-19 pandemic (2020–2022) from our sample and reran the model regressions. Table 14 presents the regression results after excluding the pandemic period. The signs of the regression coefficients remain consistent with previous findings and are statistically significant, confirming the robustness of our results.

**Table 14:** Regression after excluding COVID-19 period samples. All standard errors have been processed using White’s heteroskedasticity robust treatment and are adjusted for clustering at the firm level.

Variables	(1)	(2)	(3)
	*RPT*	*ATTEND*	*EXIT*
*DT*	-0.041*** (-3.28)	0.054*** (4.78)	0.053*** (5.86)
Control Variables	YES	YES	YES
Firm FE	YES	YES	YES
Year	YES	YES	YES
Observations	23,368	23,368	23,368
Adj-R^2^	0.145	0.380	0.377

Note:

*, ** and *** denote statistical significance at the 10%, 5%, 1% level. T-statistics are in parentheses. The control variables are the same as those in Table 4. Due to space constraints, it is not shown here.

#### 5.5.6 Redefining the scope of minority shareholders

In Section 5.4, we limited the concept of minority shareholders to shareholders beyond the top 5 shareholders of publicly listed companies when defining variables for minority shareholder attendance rates and the threat of minority shareholder withdrawal. However, definitions of the scope of minority shareholders vary across many studies, such as shareholders beyond the controlling shareholder (the largest shareholder) and those beyond the top 10 shareholders. To mitigate the impact of these definitional discrepancies on our conclusions, we redefined the scope of minority shareholders and recalculated the attendance rates and withdrawal threats of minority shareholders. Specifically, we defined two new variables, *ATTEND2* and *EXIT2*, representing the attendance rate at the annual general meeting and the exit threat of withdrawal of shareholders other than the controlling shareholder. Additionally, we defined *ATTEND3* and *EXIT3*, which represent the attendance rate and the exit threat of withdrawal for shareholders beyond the top 10 shareholders of companies. Subsequently, we reassessed these variables as alternative measures for the mechanism variables *ATTEND* and *EXIT* discussed in Section 5.4. Table 15 presents the results of the mechanism test after redefining the scope of minority shareholders. The signs of the regression coefficients remain consistent with previous findings and are statistically significant, confirming the robustness of our results.

**Table 15:** Regression after redefining the scope of minority shareholders. All standard errors have been processed using White’s heteroskedasticity robust treatment and are adjusted for clustering at the firm level.

**Variables**	**(1)**	**(2)**	**(3)**	**(4)**
	***ATTEND2***	***RPT***	***EXIT2***	***RPT***
*DT*	0.182*** (15.05)		0.032*** (4.52)	
*ATTEND2*		-0.019** (-2.32)		
*EXIT2*				-0.015** (-2.35)
Control Variables	YES	YES	YES	YES
Firm FE	YES	YES	YES	YES
Year	YES	YES	YES	YES
Observations	31,649	31,649	31,649	31,649
Adj-R^2^	0.177	0.142	0.333	0.140
**Variables**	**(5)**	**(6)**	**(7)**	**(8)**
	***ATTEND3***	***RPT***	***EXIT3***	***RPT***
*DT*	0.117*** (8.90)		0.030*** (4.26)	
*ATTEND3*		-0.024** (-2.19)		
*EXIT3*				-0.011** (-2.20)
Control Variables	YES	YES	YES	YES
Firm FE	YES	YES	YES	YES
Year	YES	YES	YES	YES
Observations	31,649	31,649	31,649	31,649
Adj-R^2^	0.195	0.141	0.341	0.138

Note:

*, ** and *** denote statistical significance at the 10%, 5%, 1% level. T-statistics are in parentheses. The control variables are the same as those in Table 4. Due to space constraints, it is not shown here.

#### 5.5.7 Regression results of various dimensions of digital transformation

During the construction of the digital transformation variable *DT* in Section 4.2.2, we integrated two aspects of digital transformation—information advantage and shareholder capability—using principal component analysis. Information advantage includes four sub-dimensions: Corporate Strategy, Financial Accounting, Infrastructure, and Business Model. Shareholder capability encompasses three sub-dimensions: Shareholder Digital Background, Fintech, and Internet Attention. To ensure the robustness of our research findings, it is necessary to conduct regression analyses of these sub-dimensions of the corporate digital transformation variable separately with the core dependent variable *RPT* and the mechanism variables *ATTEND* and *EXIT* of this study. Accordingly, we extract variables for these seven dimensions and, after standardizing them with z-score, included them in the baseline regression model and mechanism test model for re-analysis. *DT_CS* corresponds to the Corporate Strategy dimension. *DT_FA* refers to the Financial Accounting dimension. *DT_INF* denotes the Infrastructure dimension. *DT_BM* is the Business Model dimension. *DT_SDB* stands for the Shareholder Digital Background dimension. *DT_FIN* represents the Fintech dimension. *DT_WEB* is the Internet Attention dimension, derived by equally weighting and summing *DT_BAIDU* and *DT_GUBA*.

Table 16 presents the baseline regression results for *RPT* as the dependent variable and the various dimensions of digital transformation as independent variables. The table shows that all dimensions of digital transformation are significantly negatively correlated with the core dependent variable *RPT*. This is consistent with the conclusions of the baseline regression results of our study, further confirming the negative correlation between the information advantage and enhanced digital capabilities of shareholders resulting from digital transformation and the firm’s agency costs. This supports Hypothesis 1 of our study.

**Table 16:** Baseline regression results of various dimensions of digital transformation. All standard errors have been processed using White’s heteroskedasticity robust treatment and are adjusted for clustering at the firm level.

Variables	(1)	(2)	(3)	(4)	(5)	(6)	(7)
	*RPT*	*RPT*	*RPT*	*RPT*	*RPT*	*RPT*	*RPT*
*DT_CS*	-0.040*** (-4.81)						
*DT_FA*		-0.024*** (-3.14)					
*DT_INF*			-0.013*** (-3.26)				
*DT_BM*				-0.011* (-1.93)			
*DT_SDB*					-0.031** (-2.47)		
*DT_FIN*						-0.053*** (-5.09)	
*DT_WEB*							-0.010*** (-2.98)
Control Variables	YES	YES	YES	YES	YES	YES	YES
Firm FE	YES	YES	YES	YES	YES	YES	YES
Year	YES	YES	YES	YES	YES	YES	YES
Observa-tions	31,649	31,649	31,649	31,649	31,649	31,649	31,649
Adj-R^2^	0.143	0.142	0.142	0.142	0.142	0.143	0.141

Note:

*, ** and *** denote statistical significance at the 10%, 5%, 1% level. T-statistics are in parentheses. The control variables are the same as those in Table 4. Due to space constraints, it is not shown here.

Tables 17 and 18 present the baseline regression results for *ATTEND* and *EXIT* as dependent variables, respectively, across the various dimensions of digital transformation. The tables show that, except for the digitalization of the Financial Accounting dimension (*DT_FA*) and the Business Model dimension (*DT_BM*), which are not significantly related to the variable *EXIT* representing the exit threat of withdrawal by minority shareholders, other dimensions of digital transformation as independent variables are significantly positively correlated with the core mechanism variables *ATTEND* and *EXIT*. Therefore, overall, these results are consistent with our mechanism test results, further confirming the positive correlation between the information advantage and enhanced digital capabilities of shareholders resulting from digital transformation, and both the attendance rate and the threat of withdrawal by minority shareholders. This supports our Hypothesis 2. In summary, after conducting regression analyses across the various dimensions of digital transformation, our conclusions remain robust.

**Table 17:** Mechanism test results of various dimensions of digital transformation (*ATTEND* as dependent variable). All standard errors have been processed using White’s heteroskedasticity robust treatment and are adjusted for clustering at the firm level.

Variables	(1)	(2)	(3)	(4)	(5)	(6)	(7)
	*ATTEND*	*ATTEND*	*ATTEND*	*ATTEND*	*ATTEND*	*ATTEND*	*ATTEND*
*DT_CS*	0.055*** (7.39)						
*DT_FA*		0.032*** (4.83)					
*DT_INF*			0.030*** (8.25)				
*DT_BM*				0.008* (1.78)			
*DT_SDB*					0.026** (2.36)		
*DT_FIN*						0.137*** (16.93)	
*DT_WEB*							0.011*** (4.02)
Control Variables	YES	YES	YES	YES	YES	YES	YES
Firm FE	YES	YES	YES	YES	YES	YES	YES
Year	YES	YES	YES	YES	YES	YES	YES
Observa-tions	31,649	31,649	31,649	31,649	31,649	31,649	31,649
Adj-R^2^	0.377	0.374	0.376	0.373	0.373	0.386	0.380

Note:

*, ** and *** denote statistical significance at the 10%, 5%, 1% level. T-statistics are in parentheses. The control variables are the same as those in Table 4. Due to space constraints, it is not shown here.

**Table 18:** Mechanism test results of various dimensions of digital transformation (*EXIT* as dependent variable). All standard errors have been processed using White’s heteroskedasticity robust treatment and are adjusted for clustering at the firm level.

Variables	(1)	(2)	(3)	(4)	(5)	(6)	(7)
	*EXIT*	*EXIT*	*EXIT*	*EXIT*	*EXIT*	*EXIT*	*EXIT*
*DT_CS*	0.015** (2.48)						
*DT_FA*		0.007 (1.23)					
*DT_INF*			0.036*** (8.65)				
*DT_BM*				0.001 (0.12)			
*DT_SDB*					0.014** (2.38)		
*DT_FIN*						0.023*** (3.08)	
*DT_WEB*							0.008*** (3.29)
Control Variables	YES	YES	YES	YES	YES	YES	YES
Firm FE	YES	YES	YES	YES	YES	YES	YES
Year	YES	YES	YES	YES	YES	YES	YES
Observa-tions	31,649	31,649	31,649	31,649	31,649	31,649	31,649
Adj-R^2^	0.347	0.347	0.350	0.347	0.347	0.348	0.351

Note:

*, ** and *** denote statistical significance at the 10%, 5%, 1% level. T-statistics are in parentheses. The control variables are the same as those in Table 4. Due to space constraints, it is not shown here.

### 5.6 Heterogeneity tests

Given the differences in external operating environments and internal governance mechanisms among various enterprises, there are discrepancies in efficiency and cost during the digital transformation process, resulting in varying degrees of digital transformation across enterprises. To further explore and analyze the potential factors affecting the degree of digital transformation, we conduct heterogeneity tests from the perspectives of geographical regions, corporate ownership characteristics, and equity checks and balances.

#### 5.6.1 Regional differences of enterprises

Due to the variance in digital economy development levels, marketization degrees, resource endowments, and institutions across different regions in China, the activities related to digital transformation among enterprises also differ, thereby creating varied impacts on corporate governance. Specifically, compared to the central and western regions, the eastern region has a higher degree of marketization, a more developed capital market, a more optimized industry structure, and broader enterprise digital transformation. Concurrently, enterprises in the eastern region possess more rational business and management philosophies, organizational structure designs, making it easier for digital transformation to influence corporate governance. Hence, this study anticipates a greater impact of digital transformation on managerial governance in the eastern region compared to the central and western regions.

Based on the analysis above, we divided the sample into three groups: eastern region, central region, and western region, and conducted grouped regression. According to Beijing Macro-Economic and Social Development Base Database, the eastern region includes Beijing, Tianjin, Hebei, Liaoning, Shanghai, Jiangsu, Zhejiang, Fujian, Shandong, Guangdong, Guangxi, and Hainan. The central region comprises Shanxi, Nei Mongol, Jilin, Heilongjiang, Anhui, Jiangxi, Henan, Hubei, and Hunan. The western region encompasses Chongqing, Sichuan, Guizhou, Yunnan, Tibet, Shaanxi, Gansu, Ningxia, Qinghai, and Xinjiang. Columns (1)-(3) of Table 19 report the impacts of digital transformation on the governance efficiency of minority shareholders in enterprises from the eastern, central, and western regions respectively. The results indicate a negative correlation between *DT* and *RPT* in both the eastern and central regions, with a larger coefficient for *DT* in the eastern region compared to the central region. This suggests that digital transformation can reduce the extent of controlling shareholder expropriation from minority shareholders caused by related-party transactions, with this effect being more pronounced in enterprises in the eastern region. However, for the western region, the coefficient between *DT* and *RPT* is not significant, indicating no notable correlation between digital transformation and the level of related-party transactions, implying that digital transformation may not be able to influence the expropriation behavior of controlling shareholders caused by related-party transactions. A possible explanation could be the lower levels of financial development and overall digital transformation in the western region, which hinder minority shareholders from accessing internal operational information of enterprises through effective channels, thus resulting in lower enthusiasm to participate in corporate governance. Overall, the impact of digital transformation on the governance efficiency of minority shareholders exhibits a characteristic of “eastern > central > western” across different geographical regions, which is consistent with the analysis in our study.

**Table 19:** The result of heterogeneity tests. All standard errors have been processed using White’s heteroskedasticity robust treatment and are adjusted for clustering at the firm level.

**Variables**	**(1)**	**(2)**	**(3)**
	**Eastern Region**	**Central Region**	**Eastern Region**
*DT*	-0.029** (-2.03)	-0.019* (-1.83)	-0.028 (-0.81)
Control Variables	YES	YES	YES
Firm FE	YES	YES	YES
Year	YES	YES	YES
Observations	19,457	5,836	3,553
Adj-R^2^	0.146	0.123	0.134
**Variables**	**(4)**	**(5)**	
*DT*	-0.049*** (-7.23)	-0.026*** (-6.56)	
*SOE*	0.051** (2.37)		
*DT×SOE*	0.038* (1.84)		
*BALNCE*		-0.021*** (-3.22)	
*DT×BALNCE*		-0.019** (-2.15)	
Control Variables	YES	YES	
Firm FE	YES	YES	
Year	YES	YES	
Observations	31,649	31,649	
Adj-R^2^	0.142	0.143	

Note:

*, ** and *** denote statistical significance at the 10%, 5%, 1% level. T-statistics are in parentheses. The control variables are the same as those in Table 4. Due to space constraints, it is not shown here.

#### 5.6.2 The ownership attributes of enterprises

The heterogeneity in enterprise ownership can lead to significant differences in operational objectives, business environments, policy support, and business models among firms. State-owned enterprises (SOEs) are often larger in scale, have complex operational decision-making processes, and are greatly influenced by the state’s macroeconomic policies. Furthermore, executives in SOEs are primarily appointed from top to bottom by higher-level organizational departments, which minimizes the influence of minority shareholders in the governance processes of SOEs [47]. Therefore, our study anticipates that the impact of digital transformation on minority shareholder governance is lower in SOEs compared to non-state-owned enterprises (non-SOEs).

Based on the analysis above, we constructed a dummy variable for state-owned enterprises (*SOE*), where SOEs are assigned a value of 1 and non-SOEs a value of 0. We then created an interaction term *DT×SOE* between this *SOE* variable and the corporate digital transformation variable *DT*. This term was then included in regression model Eq (2) for further examination. The regression outcomes, detailed in Column (4) of Table 19, reveal that both the *SOE* moderator and the *DT×SOE* interaction term exhibit significant positive coefficients. Such results suggest that state-owned enterprises play a considerable role in buffering the adverse effects of digital transformation on related party transactions. Specifically, this pattern reveals that within non-SOEs, minority shareholders are better positioned to harness their digital capabilities and the informational gains from digital transformation. They use these benefits to mitigate the risk of interest expropriation by controlling shareholders through RPTs, reinforcing the insights of our study.

#### 5.6.3 The power balance with shareholder structure

The effectiveness of minority shareholders participating in corporate governance largely depends on the comparison of power between minority shareholders and controlling shareholders, namely, the extent of power balance with shareholder structure in listed enterprises. Specifically, for enterprises with a more dispersed shareholding structure and a higher degree of power balance, the influence of controlling shareholders is relatively weak. This creates favorable conditions for minority shareholders to actively exercise their voting rights, propose new motions, and veto old ones at shareholders’ meetings [14]. At the same time, the decision-making process in enterprises with power balance is more complex, providing conditions for minority shareholders to obtain information and act collectively. This increases the number of minority shareholders participating in shareholders’ meetings and the degree of unity, enhancing the recognizability and concentration of controlling shareholders’ tunneling behaviors, thus making controlling shareholders more cautious in their decisions to tunnel the listed enterprises through related transactions. Therefore, a higher degree of power balance helps minority shareholders play a more active role in significant shareholder meeting resolutions and the improvement of corporate governance structures by acting collectively, restraining controlling shareholders from encroaching on interests, and thus enhancing the governance efficiency of minority shareholders.

Based on the above analysis, we define the variable *BALNCE* as the measure of power balance with shareholder structure, and its calculation is the ratio of the shareholding proportions of the 2nd to 5th largest shareholders to that of the largest shareholder. Subsequently, we introduce the interaction term *DT×BALNCE* based on Eq (2). Column (5) of Table 19 reports the results of the cross-sectional difference test of power balance. In the regression results, the coefficient between *DT×BALNCE* and *RPT* is significantly negative, indicating that the increase in power balance enhances the negative marginal impact of digital transformation on the level of related transactions in enterprises. This suggests that in enterprises with a higher degree of power balance, digital transformation is more capable of reducing the agency costs caused by related transactions, thereby improving the governance effect of minority shareholders, which is consistent with our study’s analysis.

## 6. Conclusions and management implications

### 6.1 Conclusions

Our study utilizes the data from listed on the Shanghai and Shenzhen A-share market in China from 2011 to 2022 as the research sample. By analyzing publicly disclosed documents and other financial data, such as annual reports, corporate charters, prospectuses, third-party tracking rating reports from these enterprises, we reconstructed the variable for digital transformation from the perspective of information advantage and enhancement of shareholder capability brought by digital transformation, and examined the impact of digital transformation on the governance effectiveness of minority shareholders. Our conclusions encompass the following aspects:

1. The degree of digital transformation is negatively correlated with the level of related-party transactions in enterprises, suggesting that digital transformation can reduce the degree of interest conflicts between controlling shareholders and minority shareholders caused by related-party transactions.
2. The degree of digital transformation is positively correlated with the attendance rate of minority shareholders at shareholder meetings and the exit threat of minority shareholders. This implies that digital transformation, by strengthening the governance mechanisms of minority shareholders exercising voting rights and exit threats, enhances the governance efficiency of minority shareholders.
3. The impact of digital transformation on the governance effectiveness of minority shareholders exhibits heterogeneity in aspects of ownership nature, geographic region, and power balance within shareholder structure. Specifically, the inhibitory effect of digital transformation on the level of related-party transactions is stronger in non-SOEs compared to SOEs. The inhibitory effect of digital transformation on the level of related-party transactions is stronger in enterprises in the eastern region than in those in the central and western regions, with the inhibitory effect being not significant in the western region. Enterprises with a stronger power balance within the shareholder structure demonstrate a stronger inhibitory effect of digital transformation on the level of related-party transactions compared to those with a weaker power balance.

Our conclusions remain robust after a series of robustness and endogeneity tests, including Propensity Score Matching (PSM), the Heckman two-stage regression based on instrumental variables, the exogenous shock test, excluding samples from the information technology sector and COVID-19 period, redefining the scope of minority shareholders and reporting the regression results of various dimensions of digital transformation.

### 6.2 Management implications

Based on our research conclusions, we can derive the following insights for managers and policymakers:

First, for enterprise managers, it’s imperative to recognize the positive impact of digital transformation on the governance of minority shareholders. On one hand, digital transformation creates big data, giving the company an informational edge. When this data is actively disclosed, it can attract more potential minority investors. This aids in the financing and development of the company. On the other hand, digital transformation enhances the digital capabilities of minority shareholders. This enables them to better understand the company’s development status and effectively supervise controlling shareholders and senior management. Therefore, companies should further embrace digital transformation initiatives. These should utilize digital technologies not just in operational and business models but also in corporate governance. At the same time, steps can be taken to strengthen the role of minority shareholders in governance. Such steps include implementing cumulative voting systems and improving online shareholder meeting processes. It’s also vital to enhance the proxy voting rights of minority shareholders. Implementing dissenting shareholder buyback request rights is another key measure. These actions collectively ensure the lawful rights and interests of all minority shareholders. Minority shareholders should recognize the importance of actively exercising their rights and participating in corporate governance. By doing so, they can raise the overall governance level and corporate performance. This involvement also secures more investment returns.

Second, for policymakers, they need to refine corporate information disclosure systems and external governance environments. This is essential for providing practical safeguards for minority shareholders to actively exercise their rights. For example, the government can encourage companies to increase transparency in their digital transformation. This can be done through incentive mechanisms or mandated requirements. Such measures would allow investors to easily understand and track companies’ digital progress and its impact on corporate governance. Regular evaluation of policy effects, based on feedback from companies and the market, is crucial. Policymakers should adjust policies to ensure they align with actual needs. Additionally, government and related institutions can offer more digital-related education and training. This would help corporate managers and shareholders better understand and address digitalization challenges. Furthermore, our research suggests that factors like ownership nature, geographic location, and shareholder structure power balance can impact digital transformation outcomes. Therefore, policymakers should offer targeted support and guidance for different enterprise types. Actions such as promoting governance entity diversification in State-Owned Enterprises (SOEs), implementing mixed ownership reforms, and intensifying digital and capital market support in the western region are vital. Further promoting equity diversification reforms in listed companies can also be beneficial. These measures will help elevate the position of minority shareholders in the capital market, optimize corporate governance environments, and genuinely protect the lawful rights and interests of all stakeholders within enterprises.

## Supporting information

S1 File Research data and code. (ZIP)
